# Mental disorders, receipt of cardiac care following myocardial infarction and the impact of the COVID-19 pandemic: a cohort study

**DOI:** 10.1136/bmjopen-2026-124463

**Published:** 2026-09-04

**Authors:** Kelly Fleetwood, John Nolan, Colin Berry, Debbie Cavers, Stewart W Mercer, Sandosh Padmanabhan, Daniel J Smith, Robert Stewart, Amanda Vettini, Caroline A Jackson, on behalf of the CVD-COVID-UK/COVID-IMPACT Consortium

**Affiliations:** 1 Usher Institute, The University of Edinburgh, Edinburgh, UK; 2 British Heart Foundation Data Science Centre, Health Data Research UK, London, UK; 3 School of Cardiovascular and Metabolic Health, University of Glasgow, Glasgow, UK; 4 Institute of Neuroscience and Cardiovascular Research, The University of Edinburgh, Edinburgh, UK; 5 Department of Psychological Medicine, King’s College London, London, UK; 6 South London and Maudsley NHS Foundation Trust, London, UK

**Keywords:** Myocardial infarction, Quality of Health Care, COVID-19, Epidemiology

## Abstract

**Abstract:**

**Objectives:**

To compare receipt of guideline-informed myocardial infarction (MI) care by mental disorder and assess how the COVID-19 pandemic affected associations.

**Design:**

A population-based cohort study using linked electronic health records.

**Setting:**

England, November 2019 to February 2023.

**Participants:**

131 075 adults with non-ST-elevation MI (NSTEMI) and 79 045 adults with ST-elevation MI (STEMI) were identified from the Myocardial Ischaemia National Audit Project, and their prior diagnoses of mental disorder were ascertained from linked hospitalisation and primary care records.

**Outcome measures:**

We compared guideline-informed care standards for each of NSTEMI and STEMI between people with schizophrenia, bipolar disorder or depression versus those without any of these disorders. We used logistic regression to adjust for confounders and investigate differences over time.

**Results:**

Mental disorder disparities were more evident for NSTEMI than STEMI. Following NSTEMI, people with a mental disorder had lower odds of angiography eligibility and receipt, cardiac ward admission and cardiac rehabilitation referral. ORs (95% CIs) ranged from 0.25 (0.20 to 0.31) for angiography receipt for schizophrenia to 0.92 (0.89 to 0.96) for cardiac ward admission for depression. Following STEMI, people with bipolar disorder were less likely to meet the 150 min call-to-balloon target (OR 0.72; 95% CI 0.55 to 0.93), and people with schizophrenia were less likely to receive rehabilitation referral (OR 0.38; 95% CI 0.23 to 0.61) or indicated secondary prevention medication (OR 0.46, 95% CI 0.27 to 0.77). There was no clear evidence that the COVID-19 pandemic affected disparities.

**Conclusions:**

People with a mental disorder are less likely to receive guideline-informed MI care, with disparities greatest following NSTEMI and for people with schizophrenia.

**STRENGTH AND LIMITATIONS OF THIS STUDY**

- The use of a national myocardial infarction (MI) audit allowed us to identify a large cohort of non-ST-elevation MI and ST-elevation MI patients and minimise selection bias.
- Objective ascertainment of mental disorder from linked electronic health records minimised information bias.
- Our analyses adjusted for a broad range of sociodemographic, hospital admission, clinical history and MI presentation characteristics; however, some degree of residual confounding may remain.
- For our analysis of the impact of COVID-19, we could not robustly account for seasonal fluctuations since prepandemic data were only available from November 2019.

## Introduction

 Cardiovascular disease (CVD) is a major contributor to the entrenched disparity in life expectancy of people with a mental disorder compared with the general population.^1–3^ Mental disorders, including schizophrenia, bipolar disorder and depression, are associated with a higher incidence of major cardiovascular events including myocardial infarction (MI).^4 5^ Following MI, pre-existing mental disorder is associated with higher 30-day and 1-year mortality and greater risk of further vascular events.^6 7^ There is growing evidence of mental health disparities in receipt of clinical care for physical conditions, including CVD,^8^ which may partly account for differences in outcomes.

Following MI, important elements of care include early diagnosis and treatment, and access to cardiac rehabilitation and secondary prevention medication, to improve chances of recovery and survival.^9^ A recent systematic review reported lower rates of angiography, percutaneous coronary intervention (PCI) and receipt of secondary prevention medication following MI in people with, versus without, a mental disorder.^6^ However, existing studies are generally non-contemporary, have rarely reported findings separately for non-ST-elevation MI (NSTEMI) and ST-elevation MI (STEMI) and do not distinguish between specific mental disorders or focus only on a single disorder. To our knowledge, no study has comprehensively compared receipt of guideline-informed clinical care for both NSTEMI and STEMI by mental disorder in a universal healthcare setting.

Delivery of healthcare, including cardiac care, was disrupted during the COVID-19 pandemic.^10^ Reports on whether this impacted the achievement of MI care targets in the UK population are conflicting.^11 12^ To our knowledge, no study has examined the impact of the pandemic on mental disorder disparities in MI care. It is important to examine whether marginalised groups experiencing MI were disproportionately affected by the COVID-19 pandemic in order to address existing inequalities and better inform responses to future disruptions to delivery of care.

This study therefore aimed to determine (1) how MI care in England differs by mental disorder, by comparing receipt of clinical care for NSTEMI and STEMI among those with versus without schizophrenia, bipolar disorder or depression, and (2) the impact of the COVID-19 pandemic on associations between mental disorder and MI care.

## Methods

### Setting and data sources

We accessed national linked electronic health records (EHRs) from England in National Health Service England’s Secure Data Environment, made available through the British Heart Foundation Data Science Centre’s CVD-COVID-UK/COVID-IMPACT Consortium.^13^ Our primary data source was the Myocardial Ischaemia National Audit Project (MINAP), a clinical audit of patients admitted to hospital with MI.^14^ MINAP was linked to the General Practice Extraction Service Data for Pandemic Planning and Research (GDPPR) dataset. The GDPPR dataset includes patients who are currently registered and deceased patients with a date of death on or after 1 November 2019. MINAP was also linked to the Hospital Episode Statistics Admitted Patient Care (HES APC) dataset and datasets which identify COVID-19 infections (Supplementary Methods S1).

The North East-Newcastle and North Tyneside 2 research ethics committee provided ethical approval for the CVD-COVID-UK/COVID-IMPACT research programme (REC No 20/NE/0161) to access, within secure trusted research environments, unconsented, whole-population, anonymous data from EHRs collected as part of patients’ routine healthcare.

### Study design and population

Our cohort study included adults (aged ≥18 years) with a final diagnosis of NSTEMI or STEMI recorded in MINAP from 1 November 2019 to 28 February 2023 (Supplementary Methods S2). We did not include events prior to 1 November 2019, since the GDPPR dataset did not contain records for people who died before this date. We excluded patients without a linked GDPPR record and those who failed quality assurance checks. For each individual, we included the first MI within the study period.

### Mental disorders

We ascertained history of schizophrenia, bipolar disorder and depression from the GDPPR and HES APC datasets (using Systematized Nomenclature of Medicine Clinical Terms and International Classification of Diseases 10th Revision codes, respectively; Supplementary Methods S3), based on diagnoses at any time prior to each individual’s MI admission date. We categorised people according to their most severe disorder, based on an established hierarchy, with schizophrenia considered the most severe, followed by bipolar disorder and depression.^2 5 7 15^ The comparison group included people without any record of these disorders prior to their MI admission.

### Myocardial infarction care

Our outcomes were based on the quality improvement metrics reported by MINAP,^9^ which are based on national and international clinical guidelines.^16 17^ For NSTEMI, we assessed eligibility for angiography (as defined by the clinical team), receipt of angiography (among people eligible for angiography), angiography within 72 hours (among people who received angiography) and admission to a cardiac ward or coronary care unit (among people who did not die in accident and emergency). We examined the following care standards for STEMI among people who received primary PCI: call-to-balloon (CTB) time (ie, time from call for help to receipt of primary PCI) within 120 min, CTB time within 150 min, door-to-balloon (DTB) time (ie, time from arrival at hospital to receipt of primary PCI) within 60 min and DTB time within 90 min. We also assessed two outcomes relevant to both NSTEMI and STEMI among people discharged home: indicated secondary prevention medication prescribed at discharge and referral for cardiac rehabilitation (online supplemental methods S4).

### Covariates

We ascertained age, sex, deprivation (based on deciles of the Index of Multiple Deprivation,^18^ a neighbourhood-level measure of deprivation) and ethnicity from the CVD-COVID-UK/COVID-IMPACT Consortium key patient characteristics table. We defined MI admission timing variables: daytime or overnight, weekday or weekend and 4-month period during which the admission occurred, and identified the hospital where the patient was admitted (Supplementary Methods S5). We identified key clinical history variables including history of MI, history of PCI and comorbid angina, cerebrovascular disease, chronic renal failure, diabetes and heart failure from MINAP, the GDPPR dataset and the HES APC dataset (online supplemental methods S5). We ascertained MI presentation features including cardiac arrest, cardiogenic shock, ST deviation, creatinine within the first 24 hours after admission (μmol/L), heart rate on admission and the first systolic blood pressure (mm Hg) measurement after admission from the MINAP dataset. We identified patients who had COVID-19 within 14 days either side of their MI (online supplemental methods S5).

### Statistical analyses

We analysed NSTEMI and STEMI separately, using mixed-effects logistic regression to obtain ORs for the receipt of each care standard among patients with each of schizophrenia, bipolar disorder or depression versus patients without any of these disorders. We serially adjusted for groups of potential confounding factors: model 1 adjusted for age and sex, with random effects for the intercept for each hospital; model 2 additionally adjusted for timing variables; model 3 additionally adjusted for ethnicity and deprivation; model 4 additionally adjusted for clinical history; model 5 additionally adjusted for MI presentation features (online supplemental methods S6).

We investigated whether any associations between mental disorders and receipt of standards of care were affected by the COVID-19 pandemic. Since our study began in November 2019, we evaluated the effect of the pandemic by comparing associations in the prepandemic period (November 2019–February 2020) to associations in each of the following 4-month periods. We categorised time into 4-month intervals to capture fluctuations in care without making prior assumptions that specific periods (eg, lockdown periods) would have better or worse attainment of care standards. In addition to lockdowns, receipt of care may have been affected by periods of increased or decreased demand on ambulance services and hospitals. To compare the associations in the prepandemic period to those in later periods, we refitted the model using a binary mental disorder variable (any of schizophrenia, bipolar disorder or depression vs none of these disorders) since there were insufficient numbers to investigate the interaction for individual mental disorders. We then fitted a further model additionally including the interaction between the binary mental disorder variable and 4-month time period. We used a likelihood ratio test to evaluate whether the model with the interaction provided a better fit than the model without the interaction.

The primary analyses excluded patients with missing data in the outcome or any of the covariates. Missing data were minimal among sociodemographic, timing and clinical history covariates, but higher among MI presentation covariates. Hence, we conducted a post hoc sensitivity analysis based on a larger set of patients, excluding only those with missing data in the outcome or the sociodemographic, timing and clinical history covariates. This analysis included models 1 to 4 as above and tested the interaction based on model 4 (using the binary mental disorder variable).

In accordance with statistical disclosure control rules, counts are rounded to the nearest five and counts less than 10 are suppressed. We used R (V.4.1.3 and above)^19^ to conduct the statistical analysis and specifically the package lme4 (V.1.1.35.3 and above)^20^ to fit mixed-effects logistic regression models.

### Data sharing

All code lists are described in Supplementary Methods S3 and S5, and analysis code is available in our GitHub repository (https://github.com/BHFDSC/CCU046_01).

### Patient and public involvement

Our project advisory group comprised lay members and people with lived experience of mental illness and was created once funding was secured. One member also provided input during the development of the funding application. We held two advisory group meetings to provide opportunity for lay input on the proposed analyses and interpretation and discussion of study findings. These meetings also shaped the format and content of a virtual multistakeholder mini conference that we held in 2024 and plans for future dissemination.

## Results

### Descriptive findings

We included 210 120 people with an MI (online supplemental figures S1 and S2). Among 131 075 people with an NSTEMI, 875 (0.7%) had schizophrenia, 750 (0.6%) had bipolar disorder and 34 610 (26.4%) had depression. Among 79 045 people with a STEMI, 490 (0.6%) had schizophrenia, 405 (0.5%) had bipolar disorder and 19 230 (24.3%) had depression. For both NSTEMI and STEMI, there was no missing data for age, sex, admission date or time or clinical history covariates. Cardiogenic shock had the most missing data (10.9% of NSTEMI patients and 13.9% of STEMI patients). All other covariates were missing for at most 3.1% of NSTEMI patients and at most 5.8% of STEMI patients (tables 1 and 2).

**Table 1:** Baseline characteristics of patients with a Myocardial Ischaemia National Audit Project (MINAP) record of a non-ST-elevation MI (NSTEMI), by mental disorder^*^

Characteristic	Schizophrenia (n=875)	Bipolar disorder (n=750)	Depression (n=34 610)	None of these disorders (n=94 840)
Sociodemographics				
Age at MI (years), mean (SD)	64.7 (12.5)	65.3 (12.9)	67.5 (13.0)	70.6 (13.3)
Female (%)	295 (33.7)	320 (42.7)	15 170 (43.8)	27 840 (29.4)
IMD decile (%)				
1 most deprived	195 (22.3)	120 (16.0)	5210 (15.1)	9710 (10.2)
2	155 (17.7)	105 (14.0)	4155 (12.0)	9150 (9.6)
3	115 (13.1)	75 (10.0)	3830 (11.1)	9290 (9.8)
4	105 (12.0)	105 (14.0)	3655 (10.6)	9570 (10.1)
5	70 (8.0)	65 (8.7)	3470 (10.0)	9620 (10.1)
6	65 (7.4)	70 (9.3)	3275 (9.5)	9805 (10.3)
7	45 (5.1)	75 (10.0)	3100 (9.0)	9535 (10.1)
8	55 (6.3)	45 (6.0)	2870 (8.3)	9425 (9.9)
9	35 (4.0)	55 (7.3)	2645 (7.6)	9210 (9.7)
10 least deprived	30 (3.4)	40 (5.3)	2380 (6.9)	9410 (9.9)
Missing	<10 (<1.1)	<10 (<1.3)	25 (0.1)	110 (0.1)
Ethnicity				
Black	30 (3.4)	10 (1.3)	365 (1.1)	1700 (1.8)
South Asian	125 (14.3)	50 (6.7)	1785 (5.2)	7990 (8.4)
White	680 (77.7)	675 (90.0)	31 535 (91.1)	81 330 (85.8)
Mixed	<10 (<1.1)	<10 (<1.3)	240 (0.7)	685 (0.7)
Other ethnic groups	30 (3.4)	10 (1.3)	675 (2.0)	2970 (3.1)
Missing	<10 (<1.1)	<10 (<1.3)	10 (<0.1)	170 (0.2)

Location and timing				
NHS trust				
Available	855 (97.7)	725 (96.7)	33 660 (97.3)	92 260 (97.3)
Missing	20 (2.3)	25 (3.3)	955 (2.8)	2580 (2.7)
Admission day				
Week day	670 (76.6)	565 (75.3)	26 030 (75.2)	71 370 (75.3)
Weekend	205 (23.4)	190 (25.3)	8580 (24.8)	23 470 (24.7)
Admission timing				
Daytime	485 (55.4)	425 (56.7)	20 815 (60.1)	58 720 (61.9)
Overnight	390 (44.6)	330 (44.0)	13 795 (39.9)	36 120 (38.1)

Clinical history				
Previous MI (%)	235 (26.9)	215 (28.7)	9220 (26.6)	21 755 (22.9)
Previous PCI (%)	200 (22.9)	210 (28.0)	10 525 (30.4)	27 310 (28.8)
Angina (%)	420 (48.0)	390 (52.0)	17 200 (49.7)	42 710 (45.0)
Cerebrovascular disease (%)	225 (25.7)	195 (26.0)	7105 (20.5)	14 980 (15.8)
Chronic renal failure (%)	100 (11.4)	85 (11.3)	3455 (10.0)	9750 (10.3)
Diabetes (%)	430 (49.1)	320 (42.7)	13 780 (39.8)	33 475 (35.3)
Heart failure (%)	300 (34.3)	215 (28.7)	10 030 (29.0)	25 700 (27.1)

MI presentation features				
ST deviation (%)	170 (19.4)	130 (17.3)	6585 (19.0)	19 320 (20.4)
Missing (%)	30 (3.4)	15 (2.0)	1140 (3.3)	2810 (3.0)
Cardiogenic shock (%)	<10 (<1.1)	<10 (<1.3)	125 (0.4)	410 (0.4)
Missing (%)	115 (13.1)	75 (10.0)	3820 (11.0)	10 310 (10.9)
Cardiac arrest (%)	35 (4.0)	15 (2.0)	755 (2.2)	2445 (2.6)
Missing (%)	25 (2.9)	10 (1.3)	660 (1.9)	1880 (2.0)
Creatinine (μmol/L)				
Median (IQR)	83.0 (68.0, 104.0)	83.0 (68.0, 104.0)	82.0 (68.0, 101.0)	85.0 (72.0, 105.0)
Missing (%)	35 (4.0)	20 (2.7)	970 (2.8)	2475 (2.6)
SBP (mmHg)				
Mean (SD)	138.2 (27.5)	140.0 (28.2)	143.3 (27.3)	145.1 (27.4)
Missing (%)	35 (4.0)	15 (2.0)	795 (2.3)	2340 (2.5)
Heart rate (bpm)				
Mean (SD)	85.8 (20.4)	82.8 (19.8)	80.7 (19.0)	79.8 (19.2)
Missing (%)	30 (3.4)	15 (2.0)	830 (2.4)	2420 (2.6)
COVID-19 positive (%)	50 (5.7)	45 (6.0)	1715 (5.0)	4995 (5.3)

* In accordance with NHS England’s Secure Data Environment statistical disclosure control rules, counts are rounded to the nearest five and counts less than 10 are suppressed.

IMD, Index of Multiple Deprivation; MI, myocardial infarction; NHS, National Health Service; PCI, percutaneous coronary intervention.

**Table 2:** Baseline characteristics of patients with a Myocardial Ischaemia National Audit Project (MINAP) record of a ST-elevation MI (STEMI), by mental disorder^*^

Characteristic	Schizophrenia (n=490)	Bipolar disorder (n=405)	Depression (n=19 230)	None of these disorders (n=58 920)
Sociodemographics				
Age at MI (years), mean (SD)	60.3 (13.1)	61.6 (13.0)	63.6 (12.8)	66.5 (13.3)
Female (%)	130 (26.5)	170 (42.0)	7200 (37.4)	13 995 (23.8)
IMD decile (%)				
1 most deprived	95 (19.4)	65 (16.0)	3040 (15.8)	6280 (10.7)
2	85 (17.3)	45 (11.1)	2450 (12.7)	5930 (10.1)
3	70 (14.3)	50 (12.3)	2180 (11.3)	5800 (9.8)
4	60 (12.2)	45 (11.1)	2055 (10.7)	5995 (10.2)
5	45 (9.2)	40 (9.9)	1860 (9.7)	5980 (10.1)
6	30 (6.1)	20 (4.9)	1750 (9.1)	5970 (10.1)
7	30 (6.1)	40 (9.9)	1590 (8.3)	5855 (9.9)
8	30 (6.1)	50 (12.3)	1560 (8.1)	5925 (10.1)
9	25 (5.1)	25 (6.2)	1495 (7.8)	5745 (9.8)
10 least deprived	15 (3.1)	20 (4.9)	1240 (6.4)	5405 (9.2)
Missing	<10 (<2.0)	<10 (<2.5)	10 (0.1)	45 (0.1)
Ethnicity (%)				
Black	15 (3.1)	<10 (<2.5)	180 (0.9)	905 (1.5)
South Asian	50 (10.2)	15 (3.7)	825 (4.3)	4950 (8.4)
White	390 (79.6)	375 (92.6)	17 710 (92.1)	50 640 (85.9)
Mixed	10 (2.0)	<10 (<2.5)	125 (0.7)	425 (0.7)
Other ethnic groups	20 (4.1)	<10 (<2.5)	360 (1.9)	1750 (3.0)
Missing	<10 (<2.0)	<10 (<2.5)	30 (0.2)	250 (0.4)

Location and timing				
NHS trust (%)				
Available	485 (99.0%)	390 (96.3%)	18 945 (98.5%)	58 045 (98.5%)
Missing	<10 (<2.0%)	15 (3.7%)	285 (1.5%)	875 (1.5%)
Admission day (%)				
Week day	355 (72.4)	305 (75.3)	13 810 (71.8)	42 220 (71.7)
Weekend	135 (27.6)	100 (24.7)	5420 (28.2)	16 700 (28.3)
Admission timing (%)				
Daytime	265 (54.1)	225 (55.6)	11 550 (60.1)	36 845 (62.5)
Overnight	225 (45.9)	180 (44.4)	7675 (39.9)	22 075 (37.5)

Clinical history				
Previous MI (%)	85 (17.3)	65 (16.0)	3035 (15.8)	6905 (11.7)
Previous PCI (%)	315 (64.3)	285 (70.4)	14 715 (76.5)	44 735 (75.9)
Angina (%)	130 (26.5)	120 (29.6)	5885 (30.6)	14 830 (25.2)
Cerebrovascular disease (%)	80 (16.3)	80 (19.8)	2565 (13.3)	5815 (9.9)
Chronic renal failure (%)	20 (4.1)	25 (6.2)	815 (4.2)	2340 (4.0)
Diabetes (%)	190 (38.8)	145 (35.8)	6075 (31.6)	15 530 (26.4)
Heart failure (%)	180 (36.7)	115 (28.4)	6265 (32.6)	18 655 (31.7)

MI presentation features				
Cardiogenic shock (%)	25 (5.1)	15 (3.7)	580 (3.0)	2130 (3.6)
Missing (%)	75 (15.3)	40 (9.9)	2700 (14.0)	8150 (13.8)
Cardiac arrest (%)	60 (12.2)	50 (12.3)	2290 (11.9)	7630 (12.9)
Missing (%)	15 (3.1)	10 (2.5)	355 (1.8)	1330 (2.3)
Creatinine (μmol/L)				
Median (IQR)	80.0 (67.0, 99.0)	78.0 (64.2, 99.0)	78.0 (65.0, 94.0)	81.0 (69.0, 98.0)
Missing (%)	30 (6.1)	20 (4.9)	1230 (6.4)	3310 (5.6)
SBP (mmHg)				
Mean (SD)	129.3 (27.2)	128.8 (26.3)	132.7 (27.7)	133.0 (27.7)
Missing (%)	30 (6.1)	15 (3.7)	755 (3.9)	2410 (4.1)
Heart rate (bpm)				
Mean (SD)	85.2 (20.8)	82.0 (19.5)	80.1 (19.3)	79.0 (19.3)
Missing	25 (5.1)	10 (2.5)	680 (3.5)	2210 (3.8)
COVID-19 positive (%)	25 (5.1)	15 (3.7)	835 (4.3)	2595 (4.4)

* In accordance with NHS England’s Secure Data Environment (SDE) statistical disclosure control rules, counts are rounded to the nearest five and counts less than 10 are suppressed.

IMD, Index of Multiple Deprivation; MI, myocardial infarction; NHS, National Health Service; PCI, percutaneous coronary intervention; SBP, systolic blood pressure.

For both NSTEMI and STEMI, relative to the comparison group, people with each mental disorder were younger at the time of MI and were more likely to be female, live in deprived areas and have comorbidities. MI presentation features, including COVID-19 infection status, generally did not differ by mental disorder (tables 1 and 2). Relative to the comparison group, the proportion of patients receiving each NSTEMI care standard was lower in those with a mental disorder, with the lowest proportions in people with schizophrenia (table 3). Patterns of receipt of care by mental disorder for STEMI were mixed, with variation by individual disorder and across care standards (table 3).

**Table 3:** Receipt of care among people with each of NSTEMI and STEMI, by mental disorder^*^

Receipt of clinical care	Schizophrenia	Bipolar disorder	Depression	None of these disorders
NSTEMI				
Angiography eligibility (%)				
N (all patients)	875	750	34 610	94 840
Yes	690 (78.9)	610 (81.3)	28 605 (82.6)	79 290 (83.6)
No	145 (16.6)	100 (13.3)	4310 (12.5)	11 400 (12.0)
Missing	45 (5.1)	40 (5.3)	1695 (4.9)	4150 (4.4)
Angiography receipt (%)				
N (patients eligible for angiography)	690	610	28 605	79 290
Yes	490 (71.0)	510 (83.6)	24 580 (85.9)	68 575 (86.5)
No	140 (20.3)	80 (13.1)	3365 (11.8)	9325 (11.8)
Patient refused	60 (8.7)	20 (3.3)	660 (2.3)	1390 (1.8)
Angiography within 72 hours (%)				
N (patients who received angiography)	490	510	24 580	68 575
Yes	220 (44.9)	235 (46.1)	11 765 (47.9)	33 835 (49.3)
No	175 (35.7)	175 (34.3)	8230 (33.5)	22 885 (33.4)
Missing	95 (19.4)	100 (19.6)	4585 (18.7)	11 860 (17.3)
Admission to cardiac ward (%)				
N (patients who did not die in A&E)	875	750	34 595	94 750
Yes	335 (38.3)	285 (38.0)	14 595 (42.2)	40 840 (43.1)
No	510 (58.3)	440 (58.7)	19 150 (55.4)	51 555 (54.4)
Missing	25 (2.9)	25 (3.3)	845 (2.4)	2355 (2.5)
Referred for cardiac rehabilitation (%)				
N (patients discharged home and without referral for cardiac rehabilitation recorded as ‘not indicated’†)	580	480	24 300	66 345
Yes	420 (72.4)	385 (80.2)	20 265 (83.4)	55 435 (83.6)
No	80 (13.8)	45 (9.4)	2125 (8.7)	5845 (8.8)
Patient declined	20 (3.4)	10 (2.1)	380 (1.6)	820 (1.2)
Missing	55 (9.5)	40 (8.3)	1535 (6.3)	4250 (6.4)
Receipt of indicated secondary prevention medication (%)				
N (patients discharged home)	660	535	26 210	71 420
Yes	535 (81.1)	440 (82.2)	21 830 (83.3)	60 200 (84.3)
No	100 (15.2)	80 (15.0)	3630 (13.8)	9410 (13.2)
Missing	25 (3.8)	15 (2.8)	750 (2.9)	1810 (2.5)

STEMI				
Call to balloon within 120 min (%)				
N (patients who underwent primary PCI)	355	315	15 655	48 015
Yes	110 (31.0)	65 (20.6)	4475 (28.6)	14 270 (29.7)
No	190 (53.5)	210 (66.7)	9395 (60.0)	27 980 (58.3)
Missing	55 (15.5)	40 (12.7)	1785 (11.4)	5770 (12.0)
Call to balloon within 150 min (%)				
N (patients who underwent primary PCI)	355	315	15 655	48 015
Yes	175 (49.3)	140 (44.4)	8175 (52.2)	25 280 (52.7)
No	125 (35.2)	135 (42.9)	5695 (36.4)	16 965 (35.3)
Missing	55 (15.5)	40 (12.7)	1785 (11.4)	5770 (12.0)
Door to balloon within 60 min (%)				
N (patients who underwent primary PCI)	355	315	15 655	48 015
Yes	55 (15.5)	35 (11.1)	2005 (12.8)	6340 (13.2)
No	235 (66.2)	230 (73.0)	11 385 (72.7)	34 080 (71.0)
Missing	65 (18.3)	50 (15.9)	2260 (14.4)	7595 (15.8)
Door to balloon within 90 min (%)				
N (patients who underwent primary PCI)	355	315	15 655	48 015
Yes	155 (43.7)	130 (41.3)	6800 (43.4)	20 830 (43.4)
No	135 (38.0)	140 (44.4)	6595 (42.1)	19 590 (40.8)
Missing	65 (18.3)	50 (15.9)	2260 (14.4)	7595 (15.8)
Referral for cardiac rehabilitation (%)				
N (patients discharged home and without referral for cardiac rehabilitation recorded as ‘not indicated’‡)	360	320	15 965	48 720
Yes	320 (88.9)	290 (90.6)	14 805 (92.7)	44 980 (92.3)
No	25 (6.9)	15 (4.7)	490 (3.1)	1730 (3.6)
Patient declined	10 (2.8)	<10 (<3.1)	100 (0.6)	265 (0.5)
Missing	10 (2.8)	15 (4.7)	575 (3.6)	1745 (3.6)
Receipt of indicated secondary prevention medication (%)				
N (patients discharged home)	380	330	16 300	49 795
Yes	340 (89.5)	305 (92.4)	15 085 (92.5)	46 205 (92.8)
No	25 (6.6)	15 (4.5)	805 (4.9)	2525 (5.1)
Missing	15 (3.9)	<10 (<3.0)	410 (2.5)	1065 (2.1)

* In accordance with National Health Service England’s Secure Data Environment (SDE) statistical disclosure control rules, counts are rounded to the nearest five and counts less than 10 are suppressed.

† Out of NSTEMI patients discharged home, referral for cardiac rehabilitation was recorded as ‘not indicated’ for 12.9% of patients with schizophrenia, 9.3% of patients with bipolar disorder, 7.3% of patients with depression and 7.1% of patients without any of these disorders.

‡ Out of STEMI patients discharged home, referral for cardiac rehabilitation was recorded as ‘not indicated’ for 5.3% of patients with schizophrenia, <3.0% of patients with bipolar disorder, 2.1% of patients with depression and 2.2% of patients without any of these disorders.

A&E, accident and emergency; NSTEMI, non-ST-elevation MI; PCI, percutaneous coronary intervention; STEMI, ST-elevation MI.

### Mental disorder and receipt of care for non-ST-elevation myocardial infarction

**Figure 1:**
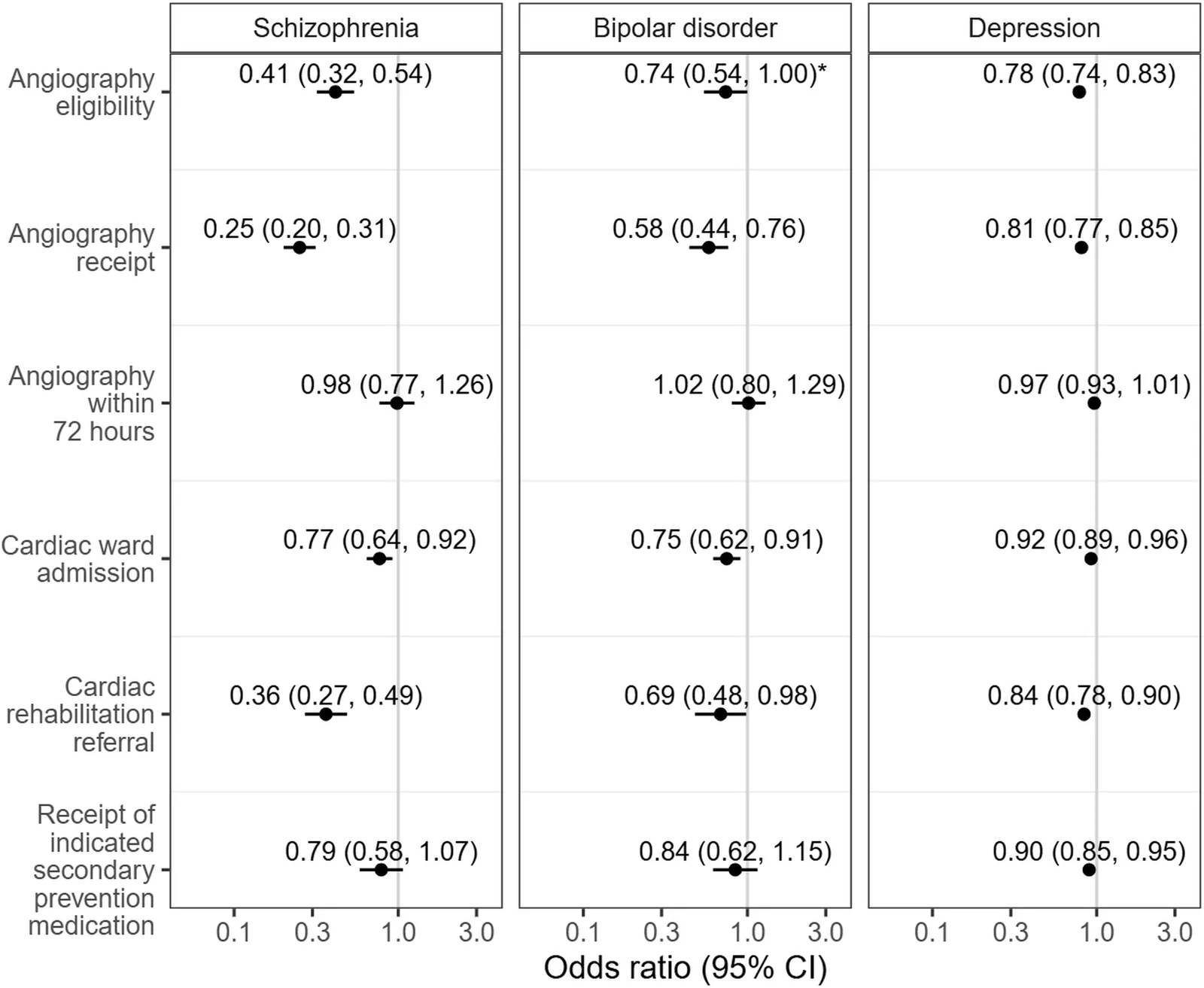
ORs for receipt of each care standard following non-ST-elevation MI (NSTEMI) in patients with schizophrenia, bipolar disorder or depression versus those without any of these disorders. ORs are adjusted for sociodemographic characteristics, hospital, myocardial infarction (MI) timing, comorbidities and MI presentation features (model 5). *0.736 (0.543, 0.998).

People with a mental disorder were less likely to receive most NSTEMI care standards, with differences largest for those with schizophrenia (figure 1). For each mental disorder, effect estimates from models one to three were similar (online supplemental figure 3), with additional adjustment for clinical history and MI presentation leading to some attenuation. Based on the fully adjusted models, mental disorder was associated with lower odds of being considered eligible for angiography (schizophrenia: OR 0.41, 95% CI 0.32 to 0.54; bipolar disorder: 0.74, 95% CI 0.54 to 1.00; depression: 0.78, 95% CI 0.74 to 0.83) and, among those eligible, to then receive angiography (schizophrenia: OR 0.25, 95% CI 0.20 to 0.31; bipolar disorder: 0.58, 95% CI 0.44 to 0.76; depression: 0.81, 95% CI 0.77 to 0.85). There was no clear difference in receipt of angiography within 72 hours by mental disorder.

People with a mental disorder were less likely to be admitted to a cardiac ward and to be referred for cardiac rehabilitation. Odds of receiving indicated secondary prevention medication were lower in people with depression than the comparison group, but there was no statistically significant difference for people with schizophrenia or bipolar disorder.

### Mental disorder and receipt of care for ST-elevation myocardial infarction

**Figure 2:**
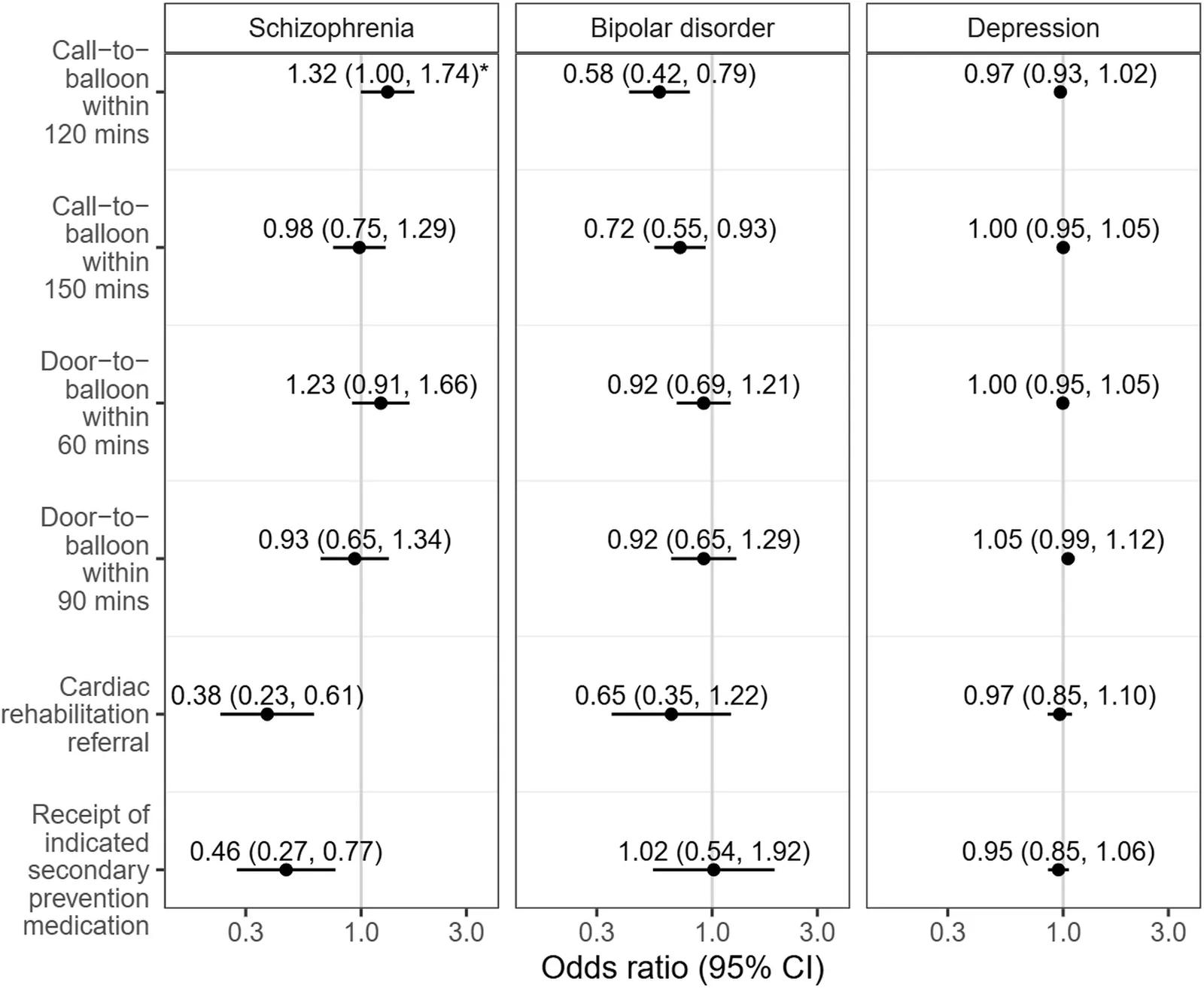
ORs for receipt of each care standard following ST-elevation MI (STEMI) in patients with schizophrenia, bipolar disorder or depression versus those without any of these disorders. ORs are adjusted for sociodemographic characteristics, hospital, myocardial infarction (MI) timing, comorbidities and MI presentation features (model 5). *1.319 (0.998, 1.744).

There was less evidence of differences in receipt of care by mental disorder for STEMI (figure 2, online supplemental figure S4). Receipt of initial reperfusion treatment was slightly lower in those with schizophrenia or bipolar disorder than in those with depression or in the comparison group (online supplemental table S1).

Bipolar disorder was associated with lower odds of meeting each of the 120 min and 150 min CTB targets (OR 0.58, 95% CI 0.42 to 0.79 and 0.72, 95% CI 0.55 to 0.93, respectively), while people with schizophrenia were less likely to be referred for cardiac rehabilitation (OR 0.38, 95% CI 0.23 to 0.61) and to receive indicated secondary prevention medication (OR 0.46, 95% CI 0.27 to 0.77). We found no other differences in receipt of care.

### Sensitivity analyses

Findings from models that included patients with complete information on sociodemographic, timing and clinical history variables but did not adjust for MI presentation features were similar to those obtained in the primary analysis for both NSTEMI and STEMI (online supplemental figures S5 and S6).

### COVID-19 and mental disorder differences in receipt of care

Within the study period, overall monthly admissions for both NSTEMI and STEMI were lowest at the start of the COVID-19 pandemic in March and April 2020 (online supplemental figure S7). For NSTEMI, the proportions of patients considered eligible for angiography and receiving angiography were lowest, while the proportion receiving angiography within 72 hours was highest in April 2020. All other care indicators were relatively stable over the study period. For STEMI, the proportions of patients meeting CTB and DTB targets decreased over the study period, whereas the proportions referred for rehabilitation and receiving appropriate secondary prevention medication were relatively stable. Time trends were generally similar for patients with versus without schizophrenia, bipolar disorder or depression (online supplemental figure S8). For both NSTEMI and STEMI, there was generally no evidence that associations between mental disorder and receipt of care varied over our study period (online supplemental results S1, online supplemental tables S2-S4).

## Discussion

Among people with NSTEMI, a history of schizophrenia, bipolar disorder or depression was associated with lower odds of angiography eligibility and receipt (if eligible), cardiac ward admission and cardiac rehabilitation referral, with the largest disparities for people with schizophrenia. Depression was additionally associated with lower receipt of secondary prevention medication. There were fewer disparities for STEMI; people with bipolar disorder were less likely to meet targets for CTB time, and those with schizophrenia had lower odds of cardiac rehabilitation referral and receipt of indicated secondary prevention medication. Within our study period, which included the acute phase of the COVID-19 pandemic and its aftermath, trends over time were similar for people with and without a mental disorder; hence, disparities were largely unaffected by the COVID-19 pandemic.

### Disparities by mental disorder

While there are consistent reports from other universal/near-universal healthcare settings of lower receipt of angiography^21–25^ and revascularisation procedures^7 21–26^ in people with mental disorders, previous studies largely focused on psychoses.^21 22 24 26^ Our findings align with and substantially add to this by identifying disparities across a wider set of guideline-indicated care standards that are evident for mood disorders as well as schizophrenia, with disparities greatest following NSTEMI. The latter is perhaps unsurprising given that NSTEMI management focuses less on rapidity of intervention, with a more staged approach to assessment, investigation, risk assessment and clinical decisions, allowing more opportunity for inequities to arise. NSTEMI disparities in angiography may be influenced by the higher prevalence of comorbidities, particularly diabetes, among patients with a mental disorder. People with diabetes experience disparities in receipt of care for MI, including reduced likelihood of receiving PCI.^27^ Given that associations persisted after adjustment for comorbidities, other factors also play a role. Our findings highlight the disparities in admission to a cardiac ward, resulting in those with a mental disorder having reduced access to dedicated, concentrated specialist nursing and medical care. Patients with NSTEMI admitted to a cardiac ward are more likely to receive guideline directed management, including angiography, PCI and optimal secondary prevention medication on discharge.^28^ Moreover, cardiac ward admission is linked to long-term prognostic benefits, including improved medication titration, early cardiac rehabilitation referral and structured discharge planning, which are thus disproportionately withheld from people with a mental disorder. We did not have information on reason for non-admission to a cardiac ward, but this may relate to delayed diagnosis among people with a mental disorder, potentially due to atypical symptom presentation or ‘diagnostic overshadowing’, whereby physical health symptoms are misattributed to mental health conditions.^29^

It is reassuring that, when PCI is conducted, DTB targets, a marker of hospital performance, did not differ by mental disorder. The lower achievement of CTB targets in people with bipolar disorder, but not schizophrenia or depression, is intriguing and implies that this subpopulation experiences delays in pre-hospital care, the reasons for which should be investigated further.

Reasons for mental disorder disparities in acute MI care have been little investigated in previous studies. However, our recent qualitative study included interviews with healthcare professionals involved in MI care provision across the acute care pathway^30^ and people with a mental disorder who had experienced an MI (*unpublished data*). We identified multiple practitioner-, patient- and system-related challenges that may impact delivery of optimal MI care. These included stigma towards patients with a mental disorder and lack of confidence in managing patient behaviour, inadequate mental health training, under-staffing, difficulties accessing input from psychiatric team colleagues, poor patient-practitioner engagement and communication, diminished patient history capacities and intervention and medication compliance concerns.

Beyond the acute treatment phase of the clinical care pathway, our study adds to existing evidence from universal healthcare settings of lower secondary prevention medication prescribing post-MI in people with a mental disorder^21 22 24 25^ by demonstrating lower likelihood of such prescribing at discharge for people with schizophrenia (following STEMI) and depression (following NSTEMI). Some medications, including beta-blockers, may be avoided due to perceived risks (eg, QT-interval prolongation).^31^ However, our findings support the need for structured documentation of contraindications to ensure optimal prescribing of vital secondary preventive medication and to ensure prescribing is not influenced by assumptions about adherence in those with a mental disorder. People with a mental disorder may be more likely to adhere to cardiovascular than psychotropic medication.^32^ Our observed disparities in cardiac rehabilitation referral are concerning. Reduced participation in cardiac rehabilitation has been linked to low socioeconomic status, smoking and history of coronary heart disease,^33^ all of which are more common in people with a mental disorder. This may lead to erroneous assumptions around cardiac rehabilitation on the part of healthcare professionals influencing referral decisions.

### Impact of the COVID-19 pandemic

While the observed mental disorder disparities in guideline-informed care are concerning, it is reassuring that the COVID-19 pandemic neither worsened nor created new disparities in MI care. This reflects other work on the impact of the COVID-19 pandemic on mental disorder disparities, which found no evidence that disparities in MI mortality^34^ and CVD incidence^15^ in England were worsened during or following the pandemic. While we found no evidence that the pandemic affected mental disorder disparities, we did find that attainment of some of the care standards varied over the course of the pandemic. The reduction in angiography receipt for the whole cohort during the first lockdown aligns with the well-documented reduction in MI hospitalisation during that time,^35^ with reduced patient burden perhaps accounting for the greater proportion with NSTEMI receiving angiogram within 72 hours. For STEMI, we observed a deterioration in the time to balloon targets over our study period, but reports from MINAP suggest this is part of a longer-term trend which predates the pandemic.^9^

### Implications

Our findings have implications for improving the delivery and clinical audit of MI care for people with a mental disorder. Greater recognition of mental disorder disparities in cardiovascular care is urgently needed.^36^ In addition to age, sex, deprivation and ethnicity, we recommend that mental disorder, often neglected, be included alongside more established sociodemographic drivers of healthcare inequalities. Mental disorder should be a stratifying variable in national cardiovascular audits and quality improvement dashboards, alongside ethnicity and deprivation. Service innovations are needed to address disparities in cardiac rehabilitation access and could include co-designed, tailored cardiac rehabilitation pathways that integrate psychiatric input, flexible schedule and peer support models.

Further research is needed to understand the reasons for observed disparities and should consider patient level factors (eg, individual preferences, MI presentation and comorbidity), practitioner level factors (eg, training and experience) and system level factors (eg, the availability of mental disorder care, including medications, and understaffing). Our qualitative study (publication forthcoming) provides much-needed insight into the experiences of, and challenges faced by, patients and healthcare professionals, but further granular quantitative *and* qualitative research is needed. Key priorities for quantitative studies include exploring detailed differences by mental disorder in the in-hospital patient journey (considering factors such as symptom presentation, time to diagnosis, ward transitions and angiogram findings), identifying factors associated with suboptimal prescribing at point of discharge and investigating access to and initiation of cardiac rehabilitation. Comparing mental disorder disparities between hospitals may help to identify individual hospitals that provide more equal care, and examining practices within any such hospitals could help to identify factors that support the care of people with a mental disorder.

### Strengths and limitations

Our study has several strengths. To our knowledge, it is the first to comprehensively investigate disparities in receipt of guideline-informed MI care by mental disorder and evaluate the impact of the COVID-19 pandemic on these disparities. The use of a national MI audit along with objective ascertainment of mental disorder from primary care records minimises both selection and information bias. The large study population allowed for separate analysis of NSTEMI and STEMI, adding valuable insights by MI type, which has rarely been addressed previously. We were also able to take account of sociodemographic factors, hospital admission factors, clinical history and MI presentation characteristics in analyses; however, some residual confounding may remain. Finally, the use of data from a contemporary time period is an important strength, given the evolution of clinical guidelines for acute MI care and increase in access to revascularisation procedures over time.

Our study has some limitations. Since we did not exclude other mental disorders from the comparison group, effect estimates may be underestimated if similar disparities are observed for people with other mental disorders. While we had planned to focus on major depression, we needed to widen our definition to depression of any severity because most depression codes used in primary care do not reflect severity. Hence, our findings for depression may not be generalisable to people with severe depression, in whom disparities may be even larger. While covariate data were mostly nearly complete, missingness was higher for MI presentation characteristics and generally higher for people with schizophrenia. Reassuringly, findings were robust to analyses adjusting for sociodemographic variables, timing and clinical history, but not MI presentation characteristics. We performed complete case analysis rather than multiple imputation given that for each outcome covariates were complete for at least 80% of participants with outcome data in the primary analyses and 97% of participants with outcome data in the sensitivity analyses where we excluded the MI presentation characteristics. Our analysis of the impact of COVID-19 was limited by complete primary care data only being available from November 2019. Thus, we could not robustly account for seasonal fluctuations in receipt of care. Finally, lower numbers of people with schizophrenia and bipolar disorder precluded investigation of interactions between individual mental disorders and time period for receipt of care.

## Conclusions

Our study found that people with schizophrenia, bipolar disorder or depression are less likely to receive guideline-informed care for MI than people without any of these disorders. Disparities were consistently observed across almost all NSTEMI care standards, for schizophrenia, bipolar disorder and depression, with findings more mixed, but with generally fewer disparities, for STEMI. Healthcare professionals should be better supported to deliver optimal MI care to patients with mental health comorbidities across the entire cardiac care pathway, from symptom assessment through to cardiac rehabilitation referral.

## Supplementary material

10.1136/bmjopen-2026-124463 online supplemental file 1

## Data Availability

Data may be obtained from a third party and are not publicly available.
