# Supplementary material for: Mental disorders, receipt of cardiac care following myocardial infarction and the impact of the COVID-19 pandemic: a cohort study
Source: BMJ Open. 2026 Sep 4;16(9):e124463. doi: 10.1136/bmjopen-2026-124463 (PMC13548243; doi:10.1136/bmjopen-2026-124463)
Supplement: Supplementary file 1 [file bmjopen-2026-124463.reviewer_comments.pdf]

PEER REVIEW HISTORY

BMJ Open publishes all reviews undertaken for accepted manuscripts. Reviewers are asked to complete a checklist review form and are provided with free text boxes to elaborate on their assessment. These free text comments are reproduced below.

ARTICLE DETAILS

Title (Provisional)

Mental disorders, receipt of cardiac care following myocardial infarction, and the impact of the COVID-19 pandemic: a cohort study

Authors

Fleetwood, Kelly; Nolan, John; Berry, Colin; Cavers, Debbie; Mercer, Stewart W; Padmanabhan, Sandosh; Smith, Daniel J.; Stewart, Robert; Vettini, Amanda; Jackson, Caroline A.; on behalf of the, CVD-COVID-UK/COVID-IMPACT Consortium

---

VERSION 1 - REVIEW

**Reviewer**	**1**
**Name**	**Reiner, Zeljko**
**Affiliation**	**Univ Zagreb, Department of internal medicine**
**Date**	**05-Jul-2026**
**COI**	**None**

This is a very interesting manuscript providing evidence that people with schizophrenia, bipolar disorder, or depression are less likely to receive guideline-informed care for MI than people without any of these disorders. The authors have correctly pointed out the limitations of their study. However, I have some comments/suggestions to the authors.

The authors did not mention anything about the drugs which the patients did receive prior to MI. Namely, it has been described that cholesterol levels might have an association with mental disorders and therefore lipid-lowering therapy might have an impact on MI outcomes. On the other hand, some psychotropic drugs could have an effect on serum lipids. Therefore, this issue has to be discussed in the Discussion, even if the authors did not have the data on treatment of the patients (the reference could be: Jakovljević M, et al. Mental disorders, treatment response, mortality and serum cholesterol: a new holistic look at old data. Psychiatr Danub. 2007 Dec;19(4):270-81).

Also, in patients with COVID-19, lipid-lowering drugs might have an beneficial effect which has been shown in a number of papers such as: Lashgari NA, et al. Statins: Beneficial Effects in Treatment of COVID-19. Adv Exp Med Biol. 2023;1412:457-476. AND Kouhpeikar H, et al. Statin Use in COVID-19 Hospitalized Patients and Outcomes: A Retrospective Study. Front Cardiovasc Med. 2022 Feb 24;9:820260.

Therefore, this should be also discussed even if the authors did not present the data on lipid-lowering treatment of patients with COVID-19 (if they have these data they should be presented and discussed).

Reviewer 2

Name Hayiroglu, Mert Ilker

Affiliation Dr. Siyami Ersek Training and Research Hospital, Department of Cardiology, Health Sciences University, Tibbiye Street, 34668, Uskudar, Istanbul, Turkey

Date 12-Jul-2026

COI None

This is a large, well-conducted national cohort study examining inequalities in guideline-informed myocardial infarction care among patients with schizophrenia, bipolar disorder, or depression in England, and evaluating whether these inequalities changed during the COVID-19 pandemic. The use of linked MINAP, primary-care, and hospital data, the separate assessment of NSTEMI and STEMI, and the analysis of multiple care indicators across the acute and post-discharge pathway are important strengths. The findings are clinically meaningful: patients with mental disorders, especially schizophrenia, had substantially lower odds of angiography eligibility and receipt, cardiac-ward admission, and referral to cardiac rehabilitation after NSTEMI, whereas disparities after STEMI were more selective. The authors should be commended for showing that these pre-existing inequalities generally persisted rather than clearly worsening during the pandemic. Nevertheless, the Discussion could be strengthened by placing the pandemic-related results in a broader context of how COVID-19 altered acute MI presentation, referral patterns, access to invasive treatment, and system-level delivery of care. In particular, the authors should clarify that the absence of strong evidence for a differential worsening of disparities by mental-disorder status does not mean that the pandemic had no important effect on MI care overall.

The authors should expand the COVID-19 section to discuss contemporary changes in MI management during the pandemic, including delayed presentation, reduced emergency attendance, altered ambulance and hospital pathways, infection-control-related delays, limited catheterisation-laboratory capacity, and reduced or delayed access to primary percutaneous coronary intervention for STEMI. They may consider citing “Effect of the COVID-19 pandemic on access to primary percutaneous coronary intervention for ST-segment elevation myocardial infarction” to provide context for changes in timely reperfusion care.

The manuscript should also acknowledge the diagnostic challenge of distinguishing acute MI from COVID-19-associated myocardial injury and myocarditis, especially when patients present with chest pain, troponin elevation, ECG abnormalities, or overlapping systemic symptoms. In this context, the authors may consider citing “COVID-19 and acute myocarditis: current literature review and diagnostic challenges.”

A short forward-looking discussion could address how future health-system disruptions should be managed to protect equitable cardiovascular care, particularly for patients with mental disorders. Potential strategies include preserving rapid triage and invasive-care pathways, strengthening integrated cardiology–mental-health services, ensuring clear referral and rehabilitation pathways, and using digital follow-up models. Mobile-health systems, remote symptom assessment, medication support, patient education, activity tracking, and digitally enabled cardiac rehabilitation may help maintain continuity of secondary prevention and rehabilitation when in-person services are disrupted. The authors may therefore consider citing "The effect of 1-year mean step count on the change in the atherosclerotic cardiovascular disease risk calculation in patients with high cardiovascular risk: a sub-study of the LIGHT randomized clinical trial" and "Digital Health Interventions in Patient Management Following Acute Coronary Syndrome: A Meta-Analysis of the Literature." These references should be framed as evidence from related high-risk cardiovascular and post-acute-coronary-syndrome populations, rather than as direct evidence in patients with mental disorders following MI.

Reviewer **3**

Name **Askin, Lütfü**

Affiliation **Gaziantep Islamic Science and Technology University, Department of Cardiology**

Date **15-Jul-2026**

COI **None**

This is a well-designed, timely, and clinically relevant nationwide cohort study addressing an important health inequality. The large sample size, use of linked national datasets, separation of NSTEMI and STEMI analyses, and assessment of the COVID-19 pandemic strengthen the manuscript. The findings are clinically meaningful and likely to influence quality improvement initiatives in cardiovascular care.

Major comments

1. The manuscript would benefit from a more detailed discussion of potential mechanisms underlying lower angiography eligibility among patients with mental disorders, particularly whether clinician decision-making, patient preference, frailty, or unmeasured confounding contributed.

2. Although extensive adjustment was performed, residual confounding (e.g., smoking, frailty, medication adherence, substance use, severity of psychiatric illness) should be discussed more explicitly as a limitation.

3. Consider expanding the clinical implications by suggesting specific interventions (integrated cardiology-psychiatry pathways, standardized referral protocols, equity-focused quality indicators) to reduce observed disparities.

Clarify whether hospital-level variation was explored beyond random intercept modelling, as institutional practice differences may partly explain disparities.

4. To improve all section add "The effect of coronavirus disease 2019 on cardiovascular diseases. Arquivos brasileiros de cardiologia, 2020, 114: 817-822.

Minor comments

Please define all abbreviations at first appearance and ensure consistency throughout the manuscript.

Report the extent and handling of missing data more prominently in the Results section.

The Discussion could briefly compare the magnitude of disparities with previous international registry studies.

Consider adding one concise figure summarizing the principal findings for NSTEMI versus STEMI to improve readability.

Carefully proofread for minor formatting inconsistencies before publication.

**

The study is methodologically sound, addresses an important clinical question, and is suitable for publication after revisions focused on improving interpretation and clinical applicability.

VERSION 1 - AUTHOR RESPONSE

Thank you for the opportunity to revise and improve our paper. Our responses to each comment are included in blue below. All page and line numbers refer to the revised track changes version of our manuscript.

Reviewer: 1

Dr. Zeljko Reiner, Univ Zagreb Comments to

the Authors:

This is a very interesting manuscript providing evidence that people with schizophrenia, bipolar disorder, or depression are less likely to receive guideline-informed care for MI than people without any of these disorders. The authors have correctly pointed out the limitations of their study. However, I have some comments/suggestions to the authors.

Authors' response: Thank you for taking the time to review our manuscript. We have responded to your suggestions below.

The authors did not mention anything about the drugs which the patients did receive prior to MI. Namely, it has been described that cholesterol levels might have an association with mental disorders and therefore lipid-lowering therapy might have an impact on MI outcomes. On the

other hand, some psychotropic drugs could have an effect on serum lipids. Therefore, this issue has to be discussed in the Discussion, even if the authors did not have the data on treatment of the patients (the reference could be: Jakovljević M, et al.
Mental disorders, treatment response, mortality and serum cholesterol: a new holistic look at old data. Psychiatr Danub. 2007 Dec;19(4):270-81).

Authors' response: We agree that the medications people receive may have an impact on cholesterol levels as well as MI outcomes such as mortality. However, our paper is focused on MI care measures, such as receipt of angiography, admission to cardiac ward and cardiac rehabilitation referral, which are not likely to be directly affected by medication use. Given that the discussion is already long, and other reviewers have also suggested additions to the discussion, we have not elaborated on medication use.

Also, in patients with COVID-19, lipid-lowering drugs might have an beneficial effect which has been shown in a number of papers such as: Lashgari NA, et al. Statins: Beneficial Effects in Treatment of COVID-19. Adv Exp Med Biol. 2023;1412:457-476. AND Kouhpeikar H, et al. Statin Use in COVID-19 Hospitalized Patients and Outcomes: A Retrospective Study. Front Cardiovasc Med. 2022 Feb 24;9:820260. Therefore, this should be also discussed even if the authors did not present the data on lipid-lowering treatment of patients with COVID-19 (if they have these data they should be presented and discussed).

Authors' response: Thank you for sharing these references which examine the potential benefits of statins in the treatment of COVID-19. As per our response above, our outcomes in this study were MI care indicators, which are unlikely to be directly affected by statin use. For this reason, and to avoid the discussion becoming unnecessarily lengthy, we have not elaborated on medication use.

Reviewer: 2

Dr. Mert Ilker Hayiroglu, Dr. Siyami Ersek Training and Research Hospital, Department of Cardiology, Health Sciences University, Tibbiye Street, 34668, Uskudar, Istanbul, Turkey

Comments to the Authors:

This is a large, well-conducted national cohort study examining inequalities in guideline-informed myocardial infarction care among patients with schizophrenia, bipolar disorder, or depression in England, and evaluating whether these inequalities changed during the
COVID-19 pandemic. The use of linked MINAP, primary-care, and hospital data, the separate assessment of NSTEMI and STEMI, and the analysis of multiple care indicators across the acute and post-discharge pathway are important strengths. The findings are clinically meaningful: patients with mental disorders, especially schizophrenia, had substantially lower odds of angiography eligibility and receipt, cardiac-ward admission, and referral to cardiac rehabilitation after NSTEMI, whereas disparities after STEMI were more selective.
The authors should be commended for showing that these pre-existing inequalities generally

persisted rather than clearly worsening during the pandemic. Nevertheless, the Discussion could be strengthened by placing the pandemic-related results in a broader context of how COVID-19 altered acute MI presentation, referral patterns, access to invasive treatment, and system-level delivery of care. In particular, the authors should clarify that the absence of strong evidence for a differential worsening of disparities by mental-disorder status does not mean that the pandemic had no important effect on MI care overall.

Authors' response: Thank you for taking the time to review our paper. In line with your suggestion, we have now signposted that the pandemic did affect the attainment of some of the care standards, see Discussion, Impact of the COVID-19 pandemic, page 20, lines 8 to 10:

Whilst we found no evidence that the pandemic affected mental disorder disparities, we did find that attainment of some of the care standards varied over the course of the pandemic.

The rest of the paragraph discusses some of the changes over the course of the pandemic.

The authors should expand the COVID-19 section to discuss contemporary changes in MI management during the pandemic, including delayed presentation, reduced emergency attendance, altered ambulance and hospital pathways, infection-control-related delays, limited catheterisation-laboratory capacity, and reduced or delayed access to primary percutaneous coronary intervention for STEMI. They may consider citing "Effect of the COVID-19 pandemic on access to primary percutaneous coronary intervention for ST-segment elevation myocardial infarction" to provide context for changes in timely reperfusion care.

Authors' response: As per our response to your first comment, we have now expanded the discussion on changes in MI care over the course of the pandemic (page 20, lines 8 to 10). This discussion refers to the trends described in our Results section (page 16, lines 15 to 21) and displayed in Figure S7. Whilst it was interesting to read about the changes in Turkey, we feel that differences in the health systems and the response to the pandemic between UK and Turkey mean that the findings in the paper cited above are not directly relevant to our discussion.

The manuscript should also acknowledge the diagnostic challenge of distinguishing acute MI from COVID-19-associated myocardial injury and myocarditis, especially when patients present with chest pain, troponin elevation, ECG abnormalities, or overlapping systemic symptoms. In this context, the authors may consider citing "COVID-19 and acute myocarditis: current literature review and diagnostic challenges."

Authors' response: Thank you for this suggestion. Our study only includes people with a final (discharge) diagnosis of NSTEMI or STEMI (see Methods > Study design and population, page 6, line 19 and Methods S2). Whilst we agree that MI and COVID-19 associated myocarditis do share some symptoms, our cohort is based on the final (discharge) diagnosis, rather than an initial diagnosis, so is less likely to be affected by misdiagnosis.

A short forward-looking discussion could address how future health-system disruptions should be

managed to protect equitable cardiovascular care, particularly for patients with mental disorders. Potential strategies include preserving rapid triage and invasive-care pathways, strengthening integrated cardiology–mental-health services, ensuring clear referral and rehabilitation pathways, and using digital follow-up models. Mobile-health systems, remote symptom assessment, medication support, patient education, activity tracking, and digitally enabled cardiac rehabilitation may help maintain continuity of secondary prevention and rehabilitation when in-person services are disrupted. The authors may therefore consider citing "The effect of 1-year mean step count on the change in the atherosclerotic cardiovascular disease risk calculation in patients with high cardiovascular risk: a sub-study of the LIGHT randomized clinical trial" and "Digital Health Interventions in Patient Management Following Acute Coronary Syndrome: A Meta-Analysis of the Literature." These references should be framed as evidence from related high-risk cardiovascular and post-acute-coronary-syndrome populations, rather than as direct evidence in patients with mental disorders following MI.

Authors' response: Thank you for your suggestion to include comments that are relevant to managing future health-system disruptions. However, given that we found no evidence that disruption from COVID-19 disproportionately affected people with a mental disorder, we feel it is more appropriate to retain the focus on implications with respect to reducing mental disorder disparities in MI care.

Reviewer: 3

Dr. Lütfü Askin, Gaziantep Islamic Science and Technology University Comments to

the Authors:

This is a well-designed, timely, and clinically relevant nationwide cohort study addressing an important health inequality. The large sample size, use of linked national datasets, separation of NSTEMI and STEMI analyses, and assessment of the COVID-19 pandemic strengthen the manuscript. The findings are clinically meaningful and likely to influence quality improvement initiatives in cardiovascular care.

Authors' response: Thank you for taking the time to review our manuscript.

Major comments

1. The manuscript would benefit from a more detailed discussion of potential mechanisms underlying lower angiography eligibility among patients with mental disorders, particularly whether clinician decision-making, patient preference, frailty, or unmeasured confounding contributed.

Authors' response: Thank you for this suggestion. Unfortunately, the mechanisms for lower angiography eligibility among patients with mental disorders are not well understood. We have highlighted in our discussion that 'Reasons for mental disorder disparities in acute MI care have been little investigated in previous studies' (page 19, lines 5-6). Based on your comment, we have

now expanded the implications to read 'Further research is needed to understand the reasons for observed disparities and should consider patient level factors (e.g. individual preferences, MI presentation and comorbidity), practitioner level factors (e.g. training and experience), and system level factors (e.g. the availability of mental disorder care, including medications, and understaffing)' (page 20, line 26 to page 21, line 2).

2. Although extensive adjustment was performed, residual confounding (e.g., smoking, frailty, medication adherence, substance use, severity of psychiatric illness) should be discussed more explicitly as a limitation.

Authors' response: Thank you for raising this important point. We have now acknowledged this as a limitation by adding 'however some residual confounding may remain' to the existing text (Discussion > Strengths and limitations > page 21, line 20)

3. Consider expanding the clinical implications by suggesting specific interventions (integrated cardiology-psychiatry pathways, standardized referral protocols, equity-focused quality indicators) to reduce observed disparities.

Authors' response: We agree that there is a need for equity-focused quality indicators and had included this on page 20 lines 21-22: "Mental disorder should be a stratifying variable in national cardiovascular audits and quality improvement dashboards, alongside ethnicity and deprivation".

In the discussion section we highlight the need for investigation and understanding of the reasons for the observed disparities (page 20, line 26 to page 21, line 9). Once the reasons for the disparities are understood then this will inform intervention approaches, which should then be evaluated. The findings from our paper do not directly allow us to draw conclusions or recommendations for specific interventions. It does however demonstrate multiple areas of inequalities across the clinical care pathway and highlights the need for subsequent research to identify the underlying reasons.

Clarify whether hospital-level variation was explored beyond random intercept modelling, as institutional practice differences may partly explain disparities.

Authors' response: Thank you for picking up on this. We did not explore hospital-level variation any further in this study. However, we have now included this as an area for future research (Discussion > Implications, page 21, lines 9 to 11):

Comparing mental disorder disparities between hospitals may help to identify individual hospitals that provide more equal care, and examining practices within any such hospitals could help to identify factors that support the care of people with a mental disorder.

4. To improve all section add "The effect of coronavirus disease 2019 on cardiovascular diseases. Arquivos brasileiros de cardiologia, 2020, 114: 817-822.

Authors' response: Thank you for sharing this reference with us which is a review of the relationship between COVID-19 and CVD, published in April 2020. Given that this review was conducted at the very beginning of the pandemic and much more evidence on the relationship

between COVID-19 and CVD has been generated since, we have decided not to include this reference.

Minor comments

Please define all abbreviations at first appearance and ensure consistency throughout the manuscript.

Authors' response: We have carefully reviewed the manuscript and ensured that all abbreviations are defined at first appearance and used consistently throughout the manuscript. Please see minor changes on pages 5 (lines 8 and 20; CVD and UK defined), 6 (lines 5-6, 9, 21-22; NHS defined, redundant abbreviation removed, sentence reworded to avoid abbreviation), 7 (line 23, IMD abbreviation added), 8 (line 9, OR abbreviation added), 17 (line 14; call-to-balloon replaced by CTB).

Report the extent and handling of missing data more prominently in the Results section.

Authors' response: We have added a more detailed description of the extent of missing data to Results > Descriptive findings (page 10, lines 2 to 5):

For both NSTEMI and STEMI, there was no missing data for age, sex, admission date or time, or clinical history covariates. Cardiogenic shock had the most missing data (10.9% of NSTEMI patients and 13.9% of STEMI patients). All other covariates were missing for at most 3.1% of NSTEMI patients, and at most 5.8% of STEMI patients (Tables 1 and 2).

The handling of missing data is described in Methods > Statistical analyses (page 9, lines 3 to 8).

The Discussion could briefly compare the magnitude of disparities with previous international registry studies.

Authors' response: Thank you for this suggestion. Given that our study examined two MI types and three mental disorders, there wasn't room to provide an in-depth comparison of the magnitude of disparities for each guideline-informed care standard, MI type and mental disorder. Instead, we have focused on giving a broad overview of the findings of previous studies, see page 18, lines 5 to 9 and page 19 lines 14 to 17.

Consider adding one concise figure summarizing the principal findings for NSTEMI versus STEMI to improve readability.

Authors' response: Figures 1 and 2 each summarise the principal findings for NSTEMI and STEMI respectively. We considered adding an additional figure as suggested, however such a figure would duplicate some of the results already included in Figures 1 and 2. As a compromise, perhaps the editor could ensure the figures are displayed near to each other to aid comparison?

Carefully proofread for minor formatting inconsistencies before publication.

Authors' response: We have carefully proofread the draft for minor formatting inconsistencies and adjusted some of the spacing around the headings and paragraphs to make sure it is consistent.

The study is methodologically sound, addresses an important clinical question, and is suitable for publication after revisions focused on improving interpretation and clinical applicability.

Authors' response: Thank you for your support of our study. We have revised the paper as per your above suggestions to improve interpretation and clinical applicability.
