# Supplementary material for: Mental disorders, receipt of cardiac care following myocardial infarction and the impact of the COVID-19 pandemic: a cohort study
Source: BMJ Open. 2026 Sep 4;16(9):e124463. doi: 10.1136/bmjopen-2026-124463 (PMC13548243; doi:10.1136/bmjopen-2026-124463)
Supplement: Supplementary file 2 [file bmjopen-16-9-s001.docx]

**Supplementary Materials**

**Contents**

[Methods S1: Additional description of data sources	3](#_Toc235534185)

[Methods S2: Diagnostic criteria for NSTEMI and STEMI	3](#_Toc235534186)

[Methods S3: Code lists for mental disorders	4](#_Toc235534187)

[Methods S4: Definition of care outcomes	5](#_Toc235534188)

[Methods S5: Additional description of variables	7](#_Toc235534189)

[Methods S6: Additional description of mixed effects logistic regression models	10](#_Toc235534190)

[Figure S1: Flow diagram for the study cohort	10](#_Toc235534191)

[Figure S2: Flow diagram for the analysis cohorts	11](#_Toc235534192)

[Figure S3: Odds ratios for receipt of each care standard following NSTEMI in patients with schizophrenia, bipolar disorder, or depression versus those without any of these disorders. Models 1 to 5.	12](#_Toc235534193)

[Table S1: Initial reperfusion treatment in patients with a MINAP record of a STEMI, by mental disorder status^a^	13](#_Toc235534194)

[Figure S4: Odds ratios for receipt of each care standard following STEMI in patients with schizophrenia, bipolar disorder, or depression versus those without any of these disorders. Models 1 to 5.	14](#_Toc235534195)

[Figure S5: Odds ratios for receipt of each care standard following NSTEMI in patients with schizophrenia, bipolar disorder, or depression versus those without any of these disorders. Sensitivity analysis. Models 1 to 4.	15](#_Toc235534196)

[Figure S6: Odds ratios for receipt of each care standard following STEMI in patients with schizophrenia, bipolar disorder, or depression versus those without any of these disorders. Sensitivity analysis. Models 1 to 4.	16](#_Toc235534197)

[Figure S7: MI admissions and receipt of care standards by month	17](#_Toc235534198)

[Figure S8: MI admissions and receipt of care standards by 4-month period and mental disorder	18](#_Toc235534199)

[Results S1: Effect of the COVID-19 pandemic on associations between mental disorders and receipt of standards of care	19](#_Toc235534200)

[Table S2: Interaction between mental disorder and 4-month time period; likelihood ratio tests of whether adding the interaction improved the fit of the model (primary analysis)	19](#_Toc235534201)

[Table S3: Odds ratios by 4-month time period for call-to-balloon within 120 mins following STEMI in patients with versus without any of schizophrenia, bipolar disorder, or depression. Model 5 with interaction between mental disorder and 4-month time period.	19](#_Toc235534202)

[Table S4: Interaction between mental disorder and 4-month time period; likelihood ratio tests of whether adding the interaction improved the fit of the sensitivity analysis models	20](#_Toc235534203)

# Methods S1: Additional description of data sources

## Myocardial Ischaemia National Audit Project (MINAP)

MINAP includes information on MI care from the call for help to discharge, patient demographics, medical history, and clinical characteristics of the MI.^1^

## General Practice Extraction Service Data for Pandemic Planning and Research (GDPPR) dataset

The GDPPR dataset captures data from 98% of general practices in England. At the time of data extraction, records were available up to 31 July 2024. Records transfer between practices when a patient moves and so should capture an individual’s entire medical history.

## Hospital Episode Statistics Admitted Patient Care (HES APC) dataset

The HES APC dataset records all inpatient admissions and day cases to NHS hospitals in England. Data were available from 1 April 1997 to 31 July 2024.

## COVID-19 datasets

We identified COVID-19 infections from the COVID-19 Hospitalisations in England Surveillance System, Second Generation Surveillance System, and Secondary Uses Service.

## References

1. Wilkinson C, Weston C, Timmis A, Quinn T, Keys A, Gale CP. The Myocardial Ischaemia National Audit Project (MINAP). *Eur Heart J Qual Care Clin Outcomes* 2020; **6**(1): 19–22.

# Methods S2: Diagnostic criteria for NSTEMI and STEMI

MINAP provides the following recommended definitions of STEMI and NSTEMI in their guide to the MINAP dataset.^1^

## STEMI

“There will normally be a history consistent with the diagnosis. The diagnosis requires the presence of cardiographic changes of ST elevation consistent with infarction of ≥2mm in contiguous chest leads and/or ST elevation of ≥1mm ST elevation in 2 or more standard leads. (New LBBB [left bundle branch block] is included.) There must be enzyme or troponin elevation. Where CK [creatine kinase] is used the peak value should exceed twice the upper limit of the reference range. Where troponin assay is used the locally accepted cut off value should be used.”

“This group includes all patients with STEMI regardless of whether typical changes were evident on the admission of ECG or developed subsequently.”

## NSTEMI

“There must be symptoms consistent with cardiac ischaemia, and there will normally be cardiographic changes consistent with this diagnosis. Troponin elevation above the locally determined reference level is mandatory.”

## References

1. National Institute for Cardiovascular Outcomes Research (NICOR). Myocardial Ischaemia National Audit Project Dataset. 2024. <https://www.nicor.org.uk/publications/data-sets/heart-attack-minap/minap-dataset-v10-3-2> (14 January 2026, date last accessed).

# Methods S3: Code lists for mental disorders

## SNOMED code lists (primary care diagnoses)

The GDPPR dataset includes a subset of all SNOMED codes used in the United Kingdom. The subset of codes included is defined by the primary care domain (PCD) reference set.^1^ The PCD reference set is also used by the Quality and Outcomes Framework (QOF) and current and historical versions of the reference set are available from NHS England.^2^ The available SNOMED codes are grouped into code clusters.^1^

To develop our SNOMED code lists for mental disorders we extracted codes from all of the clusters related to relevant mental disorder diagnoses: DEPR_COD (depression diagnosis codes), DEPRES_COD (depression resolved codes), and MH_COD (psychosis and schizophrenia and bipolar affective disease codes) from version 47·1 of the QOF cluster list (which was the most recent version at the time of developing the SMI code lists).^3^ Additionally, we mapped existing Read v2 and Clinical Terms Version 3 code lists^4^ for schizophrenia, bipolar disorder, and depression to SNOMED, and identified codes available within the GDPPR dataset. We collated all SNOMED codes identified from the clusters, and from the mapping process, and then identified codes for each of schizophrenia, bipolar disorder, or depression.

All of our SNOMED code lists are available via the HDR UK Phenotype Library:

- **Schizophrenia:** <https://phenotypes.healthdatagateway.org/phenotypes/PH1718>
- **Bipolar disorder:** <https://phenotypes.healthdatagateway.org/phenotypes/PH1719>
- **Depression:** <https://phenotypes.healthdatagateway.org/phenotypes/PH1720>

## ICD-10 code lists (hospital diagnoses)

For the HES APC dataset, we identified each mental disorder from the primary and secondary diagnosis fields, using ICD-10 codes for schizophrenia (F20, F25), bipolar disorder (F30, F31), and depression (F32, F33).

## References

1. NHS Digital. *General Practice Extraction Service (GPES) Data for Pandemic Planning and Research (GDPPR): a guide for analysts and users of the data.* 2025. <https://digital.nhs.uk/coronavirus/gpes-data-for-pandemic-planning-and-research/guide-for-analysts-and-users-of-the-data> (23 Jun 2025, date last accessed).
2. NHS Digital. *Quality and Outcomes Framework (QOF) business rules*. 2024. <https://digital.nhs.uk/data-and-information/data-collections-and-data-sets/data-collections/quality-and-outcomes-framework-qof/business-rules> (13 Jan 2025, date last accessed)
3. NHS Digital. *Quality and Outcomes Framework (QOF) business rules v47·0 2022-2023*. 2023. <https://digital.nhs.uk/data-and-information/data-collections-and-data-sets/data-collections/quality-and-outcomes-framework-qof/quality-and-outcome-framework-qof-business-rules/quality-and-outcomes-framework-qof-business-rules-v47.0-2022-2023> (13 Jan 2025, date last accessed).
4. Prigge R, Fleetwood KJ, Jackson CA, Mercer S, Kelly PAT, Sudlow C *et al*. Robustly Measuring Multiple Long-Term Health Conditions Using Disparate Linked Datasets in UK Biobank. Available at SSRN: <https://ssrn.com/abstract=4863974>.

# Methods S4: Definition of care outcomes

| MI Type | Outcome | Included patients | Outcome measure |
| --- | --- | --- | --- |
| NSTEMI | Eligibility for angiography (as defined by the clinical team; reasons for ineligibility included advanced malignancy, dementia, or progressive neurological disease) | All | Patients considered eligible for angiography (MINAP coronary angiography field [4·13] indicates that angiography was performed or is planned after discharge, that angiography was not performed or that the patient refused) |
|  | Receipt of angiography | Patients considered eligible for angiography, as defined above | Patient received angiography (MINAP coronary angiography field [4·13] indicates that angiography was performed) |
|  | Angiography within 72 hours | Patients who received angiography, as defined above | Angiography within 72 hours, where time to angiography is calculated as the number of hours from arrival at hospital (MINAP field 3·06) to local angiography (MINAP field 4·18) |
|  | Admission to cardiac ward | Patients who did not die in A&E (admission ward [MINAP field 3·17] is not recorded as ‘Died in A&E’) | Following the definition of this target in the MINAP 2022 annual report^1^, patients where admission ward (MINAP field 3·17) is recorded as ‘cardiac care unit’, ‘intensive therapy unit’, or ‘cardiac ward (non CCU)’ |
| STEMI | Call-to-balloon time within 120 minutes | Patients who underwent primary PCI. Initial reperfusion treatment (MINAP field 3·39) recorded as ‘pPCI in house’, ‘pPCI already was performed at the interventional hospital’, or ‘Referred for consideration for pPCI elsewhere’ | Call-to-balloon within 120 minutes, where call-to-balloon time is calculated as the number of minutes from the call for help (MINAP field 3·02) to reperfusion treatment (MINAP field 3·09) |
|  | Call-to-balloon time within 150 minutes |  | Call-to-balloon within 150 minutes, where call-to-balloon time is calculated as described above |
|  | Door-to-balloon time within 60 minutes |  | Door-to-balloon within 60 minutes, where door-to-balloon time is calculated as the number of minutes from arrival at hospital (MINAP field 3·06) to reperfusion treatment (MINAP field 3·09) |
|  | Door-to-balloon time within 90 minutes |  | Door-to-balloon within 90 minutes, where door-to-balloon time is calculated as described above |
| NSTEMI & STEMI | Suitable secondary prevention medication prescribed at discharge | Patients who were discharged home. Discharge destination (MINAP field 4·16) recorded as ‘Home’ | Where indicated, patient prescribed (or refused) aspirin (MINAP field 4·08), beta blocker (MINAP field 4·05), statin (MINAP field 4·07), either ACE inhibitor or angiotensin receptor antagonist (MINAP field 4·06), and either thienopyridine (MINAP field 4·27) or ticagrelor (MINAP field 4·31). For each field, we defined that the patient had prescribed (or refused) the medication where indicated if the response was recorded as ‘Yes’, ‘Contraindicated’, ‘Patient declined treatment’, ‘Not applicable’, or ‘Not indicated’. |
|  | Referral for cardiac rehabilitation | Patients who were discharged home and did not have referral for cardiac rehabilitation (MINAP field 4·09) recorded as ‘Not indicated’ (the MINAP specification indicates that cardiac rehabilitation may not be indicated due to severe co-morbidity, for example). | Referral for cardiac rehabilitation (MINAP field 4·09) recorded as ‘Yes’. |

## References

1. National Institute for Cardiovascular Outcomes Research. *National Cardiac Audit Programme. Myocardial Ischaemia National Audit Project (MINAP) 2022 Summary Report.* [https://www.nicor.org.uk/national-cardiac-audit-programme/previous-reports/heart-attack-minap-1/2022-3/nicor-minap-2022-final?layout=file](https://www.nicor.org.uk/national-cardiac-audit-programme/previous-reports/heart-attack-minap-1/2022-3/nicor-minap-2022-final?layout=file%20) (5 May 2025, date last accessed)

# Methods S5: Additional description of variables

## Sociodemographic variables

We ascertained age, sex, deprivation (based on quintiles of the Index of Multiple Deprivation, a neighbourhood-level measure of deprivation), and ethnicity from the CVD-COVID-UK/COVID-IMPACT Consortium key patient characteristics table.^1^

## Admission timing

We used the MINAP hospital arrival time variable to determine whether the admission was during the day (6am – 6pm) or overnight.

## Admission day

We used the MINAP hospital arrival time variable to determine whether the admission was on a weekday or weekend.

## 4**-month period of admission**

We used the MINAP hospital arrival time variable to identify the 4-month period during which the MI admission occurred, starting from the pre-pandemic period of November 2019 to February 2020.

## Hospital

We ascertained the hospital the patient was admitted to by linking the MINAP record to HES APC records.

## Comorbid angina

We identified comorbid angina if any of the following occurred:

- The MINAP previous angina field (field 2·06) recorded “1. Yes”
- The patient had a diagnosis of angina recorded in GDPPR prior to their date of MI admission.
- The patient had a diagnosis of angina recorded in HES APC prior to their date of MI admission.

We identified diagnoses using existing SNOMED (GDPPR) and ICD-10 (HES APC) code lists for angina which are available on the HDR UK Phenotype Library.^2^

## Comorbid cerebrovascular disease

We identified comorbid cerebrovascular disease if any of the following occurred:

- The MINAP cerebrovascular disease field (field 2·10) recorded “1. Yes”
- The patient had a diagnosis of cerebrovascular disease recorded in GDPPR prior to their date of MI admission. We identified diagnoses using SNOMED codes in the haemorrhagic stroke (HSTRK_COD), non-haemorrhagic stroke (OSTR_COD), stroke diagnosis (STRK_COD), and transient ischaemic attack (TIA_COD) code clusters (see Methods S2 for further explanation of code clusters) and additionally the SNOMED codes 195163003, 67992007 and 73192008.
- The patient had a diagnosis of cerebrovascular disease recorded in HES APC prior to their date of MI admission. We identified diagnoses using ICD-10 codes I60-I69 and G45.

Our code lists for comorbid cerebrovascular disease are available on our GitHub repository (<https://github.com/BHFDSC/CCU046_01>) and via the HDR UK Phenotype Library: <https://phenotypes.healthdatagateway.org/phenotypes/PH1791>.

## Comorbid chronic renal failure

We identified comorbid chronic renal failure if any of the following occurred:

- The MINAP chronic renal failure field (field 2·12) recorded “1. Yes”
- The patient had a diagnosis of chronic renal failure recorded in GDPPR prior to their date of MI admission. We identified diagnoses using SNOMED codes. We developed the code list by identifying chronic renal failure codes from an existing chronic kidney disease code list^4^ and the GDPPR code cluster for chronic kidney disease stage 4 and 5 (CKDATRISK1_COD).
- The patient had a diagnosis of chronic renal failure recorded in HES APC prior to their date of MI admission. We identified diagnoses using ICD-10 codes. We developed the code list by identifying chronic renal failure codes from an existing chronic kidney disease code list^3^ and existing end stage renal disease code lists.^4,5^

Our code lists for comorbid chronic renal failure are available on our GitHub repository (<https://github.com/BHFDSC/CCU046_01>) and via the HDR UK Phenotype Library: <https://phenotypes.healthdatagateway.org/phenotypes/PH1792>.

## Comorbid diabetes

We identified comorbid diabetes if any of the following occurred:

- The MINAP diabetes field (field 2·17) recorded "1. Diabetes (dietary control)" or "2. Diabetes (oral medicine)" or "3. Diabetes (insulin)" or "5. Insulin plus oral medication"
- The patient had a diagnosis of diabetes recorded in GDPPR prior to their date of MI admission.
- The patient had a diagnosis of diabetes recorded in HES APC prior to their date of MI admission.

We identified diagnoses using existing SNOMED (GDPPR) and ICD-10 (HES APC) code lists for diabetes which are available on the HDR UK Phenotype Library.^6^

## Comorbid heart failure

We identified comorbid heart failure if any of the following occurred:

- The MINAP heart failure field (field 2·13) recorded “1. Yes”
- The patient had a diagnosis of heart failure recorded in GDPPR prior to their date of MI admission.
- The patient had a diagnosis of heart failure recorded in HES APC prior to their date of MI admission.

We identified diagnoses using existing SNOMED (GDPPR) and ICD-10 (HES APC) code lists for heart failure which are available on the HDR UK Phenotype Library.^7^

## Previous MI

We identified previous MI if either of the following occurred:

- The patient had a diagnosis of MI recorded in GDPPR more than 3 days prior to their date of MI admission (to ensure that we didn’t count the index event). We identified diagnoses by adapting an existing MI SNOMED code list.^8^
- The patient had a diagnosis of MI recorded in HES APC more than 3 days prior to their date of MI admission. We identified diagnoses using ICD-10 codes (I21, I22) as per previous studies from the authors.^9^

Our code lists for myocardial infarction are available on our GitHub repository (<https://github.com/BHFDSC/CCU046_01>) and via the HDR UK Phenotype Library (<https://phenotypes.healthdatagateway.org/phenotypes/PH1722>).

We couldn’t use the MINAP previous MI field (field 2·05) because it wasn’t included in the version of the MINAP dataset held by the CVD-COVID-UK/COVID-IMPACT Consortium at the time of data curation.

## Previous PCI

We identified previous PCI if any of the following occurred:

- The MINAP previous PCI field (field 2·18) recorded “1. Yes”
- The patient had a PCI recorded in HES APC prior to their date of MI admission. We identified PCI using the OPCS-4 procedure code K75, in line with previous studies from the authors.^9^

We didn’t use GDPPR to identify PCI because the GDPPR clusters do not include a comprehensive set of PCI codes.

## COVID-19 positive

We identified patients who had COVID-19 within 14 days either side of their MI admission based on diagnoses recorded in GDPPR or HES APC, hospitalised cases of COVID-19 recorded in COVID-19 Hospitalisations in England Surveillance System (CHESS), and positive tests for COVID-19 recorded in Second Generation Surveillance System (SGSS) or Secondary Uses Service (SUS).

## References

1. British Heart Foundation Data Science Centre. BHF DSC HDS Documentation. Key Patient Characteristics. 2025. <https://bhfdsc.github.io/documentation/curated_assets/kpcs> (24 April 2025, date last accessed).
2. Knight R, Walker V, Ip S, Cooper JA, Bolton T, Keene S et al. *PH956 / 2134 - CCU002_01 Angina.* 2022. [http://phenotypes.healthdatagateway.org/phenotypes/PH956/version/2134/detail/](http://phenotypes.healthdatagateway.org/phenotypes/PH956/version/2134/detail/%20) (24 April 2025, date last accessed).
3. Knight R, Walker V, Ip S, Cooper JA, Bolton T, Keene S et al. *PH950 / 2128 - CCU002_01 Chronic Kidney Disease (CKD).* 2022. <http://phenotypes.healthdatagateway.org/phenotypes/PH950/version/2128/detail/> (25 April 2025, date last accessed).
4. Mansfield KE, Nitsch D, Smeeth L, Bhaskaram K, Tomlinson LA. *PH574 / 1148 - End-Stage Renal Disease (ESRD).* 2021. <http://phenotypes.healthdatagateway.org/phenotypes/PH574/version/1148/detail/> (25 April 2025, date last accessed).
5. Prigge R, Fleetwood KJ, Jackson CA, Mercer S, Kelly PAT, Sudlow C *et al*. Robustly Measuring Multiple Long-Term Health Conditions Using Disparate Linked Datasets in UK Biobank. Available at SSRN: <https://ssrn.com/abstract=4863974>.
6. Wood A, Denholm R, Hollings S, Cooper J, Ip S, Walker V et al. *PH945 / 2123 - CCU000 Diabetes.* 2022. <http://phenotypes.healthdatagateway.org/phenotypes/PH945/version/2123/detail/> (25 April 2025, date last accessed).
7. Knight R, Walker V, Ip S, Cooper JA, Bolton T, Keene S et al. *PH968 / 2146 - CCU002_01 Heart failure.* 2022. [http://phenotypes.healthdatagateway.org/phenotypes/PH968/version/2146/detail/](http://phenotypes.healthdatagateway.org/phenotypes/PH968/version/2146/detail/.%20) (25 April 2025, date last accessed).
8. Wood A, Denholm R, Hollings S, Cooper J, Ip S, Walker V et al. *PH942 / 2120 - CCU000 Acute Myocardial Infarction (AMI)*. 2022. <http://phenotypes.healthdatagateway.org/phenotypes/PH942/version/2120/detail/> (25 April 2025, date last accessed).
9. Fleetwood K, Wild SH, Smith DJ, Mercer SW, Licence K, Sudlow CLM, et al. Severe mental illness and mortality and coronary revascularisation following a myocardial infarction: a retrospective cohort study. *BMC Med* 2021;**19**:67. doi: <https://doi.org/10.1186/s12916-021-01937-2>

# Methods S6: Additional description of mixed effects logistic regression models

We serially adjusted for groups of potential confounding factors:

- **model 1** adjusted for age and sex, with random effects for the intercept for each hospital;
- **model 2** additionally adjusted for timing variables;
- **model 3** additionally adjusted for ethnicity and deprivation;
- **model 4** additionally adjusted for clinical history (history of MI, history of PCI, and comorbidities); and
- **model 5** additionally adjusted for MI presentation features (cardiac arrest, cardiogenic shock, creatinine, heart rate, SBP, and COVID-19 status for both MI types, and additionally ST deviation for NSTEMI only)

Age, creatinine, heart rate, and SBP were included in the models as continuous variables. We scaled age, SBP, and heart rate by subtracting their respective means and dividing by their standard deviations. Creatinine was right skewed and was therefore log transformed prior to scaling to improve model convergence. For all continuous variables, we included both linear and quadratic terms in the models because exploratory plots suggested that quadratic functions were sufficient for describing the relationships between these variables and the outcomes.

# Figure S1: Flow diagram for the study cohort

# Figure S2: Flow diagram for the analysis cohorts

Cohort of people with NSTEMI or STEMI

N = 210 120

Secondary prevention medication^f^

N = 66 810

N^1^ = 54 625 (81·8%)

N^2^ = 64 315 (96·3%)

Cardiac rehabilitation referral^e^

N = 65 365

N^1^ = 52 340 (80·1%)

N^2^ = 62 080 (95·0%)

Door-to-balloon time^g^

N = 64 345

N^1^ = 43 980 (68·4%)

N^2^ = 53 555 (83·2%)

Call-to-balloon time^g^

N = 64 345

N^1^ = 45 975 (71·5%)

N^2^ = 55 805 (86·7%)

^a^ N = patients eligible for inclusion in analysis

N^1^ = complete cases (with percentage of N)

N^2^ = complete cases (sensitivity analyses) (with percentage of N)

^b^ Among those considered eligible for an angiogram

^c^ Among those who received an angiogram

^d^ Among those who did not die in A&E

^e^ Among those discharged home who did not have referral for cardiac rehabilitation recorded as ‘Not indicated’

^f^ Among those discharged home

^g^ Among those who underwent primary PCI

As per NHSE’s SDE statistical disclosure control rules, counts are rounded to the nearest 5 and counts less than 10 are suppressed

Secondary prevention medication^f^

N = 98 830

N^1^ = 80 245 (81·2%)

N^2^ = 93 420 (94·5%)

Cardiac rehabilitation referral^e^

N = 91 710

N^1^ = 71 010 (77·4%)

N^2^ = 83 450 (91·0%)

Cardiac ward admission^d^

N = 130 970

N^1^ = 106 120 (81·0%)

N^2^ = 124 040 (94·7%)

Angiogram within 72 hours^c^

N = 94 155

N^1^ = 65 575 (69·6%)

N^2^ = 75 470 (80·2%)

Receipt of angiogram^b^

N = 109 200

N^1^ = 90 595 (83·0%)

N^2^ = 106 105 (97·2%)

Angiogram eligibility

N = 131 075

N^1^ = 103 595 (79·0%)

N^2^ = 121 420 (92·6%)

People with NSTEMI^a^

N = 131 075

People with STEMI^a^

N = 79 045

# Figure S3: Odds ratios for receipt of each care standard following NSTEMI in patients with schizophrenia, bipolar disorder, or depression versus those without any of these disorders. Models 1 to 5.

Model 1 adjusts for age, sex, and hospital (random effect); model 2 additionally adjusts for timing variables; model 3 adds ethnicity and area-level deprivation; model 4 adds clinical history; and model 5 adds MI presentation features.

^a^ 0·736 (0·543, 0·998)

# Table S1: Initial reperfusion treatment in patients with a MINAP record of a STEMI, by mental disorder status^a^

| Characteristic | Schizophrenia  (N = 490) | Bipolar disorder  (N = 405) | Depression  (N = 19 230) | None of these disorders  (N = 58 920) |
| --- | --- | --- | --- | --- |
| **Initial reperfusion treatment** |  |  |  |  |
| pPCI in house | 340 (69·4%) | 310 (76·5%) | 15075 (78·4%) | 46400 (78·8%) |
| pPCI already performed at interventional hospital | <10 (<2·0%) | <10 (<2·5%) | 260 (1·4%) | 680 (1·2%) |
| Referred for consideration for pPCI elsewhere | 15 (3·1%) | <10 (<2·5%) | 315 (1·6%) | 935 (1·6%) |
| Thrombolytic treatment | <10 (<2·0%) | <10 (<2·5%) | 60 (0·3%) | 125 (0·2%) |
| None | 120 (24·5%) | 85 (21·0%) | 3340 (17·4%) | 10210 (17·3%) |
| Missing | 10 (2·0%) | <10 (<2·5%) | 175 (0·9%) | 570 (1·0%) |
| **Reason treatment not given amongst patients where initial reperfusion treatment was recorded as ‘None’** |  |  |  |  |
| N | 120 | 85 | 3340 | 10 210 |
| Ineligible ECG | 15 (12·5%) | <10 (<11·8%) | 350 (10·5%) | 1015 (9·9%) |
| Too late | 40 (33·3%) | 30 (35·3%) | 1020 (30·5%) | 3325 (32·6%) |
| Risk of haemorrhage | <10 (<8·3%) | <10 (<11·8%) | 25 (0·7%) | 50 (0·5%) |
| Uncontrolled hypertension | <10 (<8·3%) | <10 (<11·8%) | <10 (<0·3%) | <10 (<0·1%) |
| Administrative failure | <10 (<8·3%) | <10 (<11·8%) | 15 (0·4%) | 40 (0·4%) |
| Elective decision | 35 (29·2%) | 20 (23·5%) | 895 (26·8%) | 2485 (24·3%) |
| Patient refused treatment | <10 (<8·3%) | <10 (<11·8%) | 55 (1·6%) | 110 (1·1%) |
| None | <10 (<8·3%) | <10 (<11·8%) | 115 (3·4%) | 415 (4·1%) |
| Other | 20 (16·7%) | 15 (17·6%) | 690 (20·7%) | 2175 (21·3%) |
| Missing | <10 (<8·3%) | <10 (<11·8%) | 165 (4·9%) | 590 (5·8%) |

pPCI: primary percutaneous coronary intervention

^a^In accordance with NHSE’s SDE statistical disclosure control rules, counts are rounded to the nearest 5 and counts less than 10 are suppressed

# Figure S4: Odds ratios for receipt of each care standard following STEMI in patients with schizophrenia, bipolar disorder, or depression versus those without any of these disorders. Models 1 to 5.

Model 1 adjusts for age, sex, and hospital (random effect); model 2 additionally adjusts for timing variables; model 3 adds ethnicity and area-level deprivation; model 4 adds clinical history; and model 5 adds MI presentation features.

^a^ 1·319 (0·998, 1·744)

^b^ 1·061 (0·997, 1·130)

# Figure S5: Odds ratios for receipt of each care standard following NSTEMI in patients with schizophrenia, bipolar disorder, or depression versus those without any of these disorders. Sensitivity analysis. Models 1 to 4.

Model 1 adjusts for age, sex, and hospital (random effect); model 2 additionally adjusts for timing variables; model 3 adds ethnicity and area-level deprivation; and model 4 adds clinical history.

# Figure S6: Odds ratios for receipt of each care standard following STEMI in patients with schizophrenia, bipolar disorder, or depression versus those without any of these disorders. Sensitivity analysis. Models 1 to 4.

Model 1 adjusts for age, sex, and hospital (random effect); model 2 additionally adjusts for timing variables; model 3 adds ethnicity and area-level deprivation; and model 4 adds clinical history.

^a^ 1·055 (0·996, 1·117)

^b^ 0·909 (0·829, 0·996)

# Figure S7: MI admissions and receipt of care standards by month

# Figure S8: MI admissions and receipt of care standards by 4-month period and mental disorder

# Results S1: Effect of the COVID-19 pandemic on associations between mental disorders and receipt of standards of care

For both NSTEMI and STEMI, there was no evidence that associations between mental disorder and receipt of care varied over our study period, with one exception (Table S2). For STEMI, the association between mental disorder and CTB within 120 minutes varied across the study period. There was a statistically significant difference between the associations in the pre-pandemic period (November 2019 – February 2020), and the association in July 2021 – October 2021; however, neither association indicated a difference between people with versus without mental disorder (Table S3). For the sensitivity analysis, for both NSTEMI and STEMI, there was no evidence that associations between mental disorder and receipt of care varied over our study period (Table S4).

# Table S2: Interaction between mental disorder and 4-month time period; likelihood ratio tests of whether adding the interaction improved the fit of the model (primary analysis)

| MI type | Care standard | $\chi_{9}^{2}$ | p-value |
| --- | --- | --- | --- |
| NSTEMI | Angiography eligibility | 13·424 | 0·144 |
|  | Angiography receipt | 5·414 | 0·797 |
|  | Angiography within 72 hrs | 8·560 | 0·479 |
|  | Admission to cardiac ward | 2·363 | 0·984 |
|  | Referral for cardiac rehabilitation | 6·281 | 0·712 |
|  | Receipt of all secondary prevention medication | 13·218 | 0·153 |
| STEMI | Call to balloon within 120 mins | 18·171 | 0·033 |
|  | Call to balloon within 150 mins | 8·006 | 0·534 |
|  | Door to balloon within 60 mins | 14·734 | 0·099 |
|  | Door to balloon within 90 mins | 10·147 | 0·339 |
|  | Referral for cardiac rehabilitation | 4·075 | 0·906 |
|  | Receipt of all secondary prevention medication | 6·733 | 0·665 |

# Table S3: Odds ratios by 4-month time period for call-to-balloon within 120 mins following STEMI in patients with versus without any of schizophrenia, bipolar disorder, or depression. Model 5 with interaction between mental disorder and 4-month time period.

| 4-month time period | Odds ratio (95% confidence interval) |
| --- | --- |
| Nov 2019 - Feb 2020 | 0·92 (0·80, 1·06) |
| Mar 2020 - Jun 2020 | 0·97 (0·84, 1·13) |
| Jul 2020 - Oct 2020 | 1·01 (0·87, 1·16) |
| Nov 2020 - Feb 2021 | 0·85 (0·73, 0·98) |
| Mar 2021 - Jun 2021 | 0·93 (0·81, 1·06) |
| Jul 2021 - Oct 2021 | 1·15 (0·99, 1·35) |
| Nov 2021 - Feb 2022 | 1·11 (0·95, 1·29) |
| Mar 2022 - Jun 2022 | 1·00 (0·85, 1·16) |
| Jul 2022 - Oct 2022 | 1·03 (0·88, 1·21) |
| Nov 2022 - Feb 2023 | 0·81 (0·68, 0·95) |

# Table S4: Interaction between mental disorder and 4-month time period; likelihood ratio tests of whether adding the interaction improved the fit of the sensitivity analysis models

| MI type | Care standard | $\chi_{9}^{2}$ | p-value |
| --- | --- | --- | --- |
| NSTEMI | Angiography eligibility | 13·076 | 0·159 |
|  | Angiography receipt | 6·143 | 0·726 |
|  | Angiography within 72 hrs | 6·234 | 0·716 |
|  | Admission to cardiac ward | 2·421 | 0·983 |
|  | Referral for cardiac rehabilitation | 3·408 | 0·946 |
|  | Receipt of all secondary prevention medication | 10·922 | 0·281 |
| STEMI | Call to balloon within 120 mins | 9·678 | 0·377 |
|  | Call to balloon within 150 mins | 6·409 | 0·698 |
|  | Door to balloon within 60 mins | 14·482 | 0·106 |
|  | Door to balloon within 90 mins | 10·608 | 0·304 |
|  | Referral for cardiac rehabilitation | 4·465 | 0·878 |
|  | Receipt of all secondary prevention medication | 10·861 | 0·285 |
